# Genome-Wide Association Study of Treatment Refractory Schizophrenia in Han Chinese

**DOI:** 10.1371/journal.pone.0033598

**Published:** 2012-03-27

**Authors:** Ying-Jay Liou, Hui-Hung Wang, Ming-Ta Michael Lee, Sheng-Chang Wang, Hung-Lun Chiang, Cheng-Chung Chen, Ching-Hua Lin, Ming-Shun Chung, Chien-Cheng Kuo, Ding-Lieh Liao, Ching-Kuan Wu, Chih-Min Liu, Yu-Li Liu, Hai-Gwo Hwu, I-Ching Lai, Shih-Jen Tsai, Chia-Hsiang Chen, Hui-Fen Liu, Yi-Chun Chou, Chien-Hsiun Chen, Yuan-Tsong Chen, Chen-Jee Hong, Jer-Yuarn Wu

**Affiliations:** 1 Department of Psychiatry, Taipei Veterans General Hospital, Taipei, Taiwan; 2 Institute of Clinical Medicine, National Yang-Ming University, Taipei, Taiwan; 3 Institute of Biomedical Sciences, Academia Sinica, Taipei, Taiwan; 4 School of Chinese Medicine, China Medical University, Taichung, Taiwan; 5 Division of Mental Health and Addiction Medicine, Institute of Population Health Sciences, National Health Research Institutes, Zhunan, Taiwan; 6 Kaohsiung Kai-Suan Psychiatric Hospital, Kaohsiung, Taiwan; 7 Jianan Mental Hospital, Department of Health, Executive Yuan, Taiwan; 8 Bali Psychiatric Center, Department of Health, Executive Yuan, Taiwan; 9 Tsyr-Huey Mental Hospital, Kaohsiung, Taiwan; 10 Department of Psychiatry, National Taiwan University Hospital and National Taiwan University College of Medicine, Taipei, Taiwan,; 11 Department of Psychology, College of Science, National Taiwan University, Taipei, Taiwan; 12 Yuli Veterans Hospital, Hualien, Taiwan; 13 Department of Medicine, National Yang-Ming University, Taipei, Taiwan; 14 Department of Pediatrics, Duke University Medical Center, Durham, North Carolina, United States of America; 15 Institute of Brain Science, National Yang-Ming University, Taipei, Taiwan; Baylor College of Medicine, United States of America

## Abstract

We report the first genome-wide association study of a joint analysis using 795 Han Chinese individuals with treatment-refractory schizophrenia (TRS) and 806 controls. Three loci showed suggestive significant association with TRS were identified. These loci include: rs10218843 (*P* = 3.04×10^−7^) and rs11265461 (*P* = 1.94×10^−7^) are adjacent to signaling lymphocytic activation molecule family member 1 (*SLAMF1*); rs4699030 (*P* = 1.94×10^−6^) and rs230529 (*P* = 1.74×10^−7^) are located in the gene nuclear factor of kappa light polypeptide gene enhancer in B-cells 1 (*NFKB1*); and rs13049286 (*P* = 3.05×10^−5^) and rs3827219 (*P* = 1.66×10^−5^) fall in receptor-interacting serine/threonine-protein kinase 4 (*RIPK4*). One isolated single nucleotide polymorphism (SNP), rs739617 (*P* = 3.87×10^−5^) was also identified to be associated with TRS. The -94delATTG allele (rs28362691) located in the promoter region of *NFKB1* was identified by resequencing and was found to associate with TRS (*P* = 4.85×10^−6^). The promoter assay demonstrated that the -94delATTG allele had a significant lower promoter activity than the -94insATTG allele in the SH-SY5Y cells. This study suggests that rs28362691 in *NFKB1* might be involved in the development of TRS.

## Introduction

Schizophrenia is a severe psychiatric disorder with a prevalence estimated to be approximately 1% [1] in the world and 0.6% in Taiwan [2]. It is the third-leading cause of disability among individuals age between 15 and 44 [3]. Its clinical manifestations are characterized by distortion of reality, delusions, hallucinations, altered emotional reactivity, disorganized behavior, social isolation and cognitive impairment. The etiology of schizophrenia is not well understood but it has been postulated as a complex disease with an estimated heritability as high as 80% [4] [5].

Genetic studies based on linkage and positional candidate genes approaches have suggested multiple candidate molecules in the pathogenesis of schizophrenia, including the receptors of antipsychotics (*DRD2* [6], *HTR2A* [7], *CHRNA7* [8], *TAAR6* [9]); the enzymes affecting neurotransmitter metabolisms (*COMT* [10], *DAOA* [11]), factors involved in microtubules function (*DISC1* [12]), neuronal differentiation (*NRG1* [13]), signal transduction (*RGS4* [14]) and calmodulin-dependent protein phosphatase (*PPP3CC* [15]). However, most of these genes lack of replicable support across populations [16], [17].

Genome-wide association study (GWAS) is a hypothesis-free approach to comprehensively identify disease susceptibility loci. It has identified several susceptible genetic variants associated with schizophrenia, such as SNPs located on or near genes involved in transcriptional regulations (*ZNF804A* [18], [19] and *ZNF184* [20]); neuronal functioning (*NRGN* [21], and *ANK3* [22]); cytokine activities (*CSF2RA* [23] and *IL3RA* [23]), inflammatory responses (*PLAA* [22]), immune function (MHC region [20], [21], [24] and *TCF4* [21]); brain development (*RPGRIP1L* [18], *PLXNA2* [25], *RELN* [26]); endocrine function (*ACSM1* [22]); and chromatin remodeling (*SMARCA2* [27]). However, these studies have not replicated the candidate genes or linkage studies in schizophrenia and most of the findings from GWAS are still inconsistent. The discordant results were likely due to the phenotypic variability associated with schizophrenia since schizophrenia is a heterogeneous disorder as well as the lack of statistical power due to find common variants of susceptibility.

Antipsychotic medication is the major treatment for schizophrenia. However, one fifth to one third of schizophrenic patients do not respond to antipsychotic treatments [28], [29]. These patients with treatment refractory schizophrenia (TRS) have persistent psychotic symptoms combining with poor social/work function in spite of administering at least two trials of sufficient antipsychotic doses and adequate treatment duration [28]. Comparing with those patients with adequate responses to antipsychotic treatments, patients with TRS had significantly lower levels of catecholamine in cerebrospinal fluid or plasma [30], increased cortical atrophy [31], [32], and a lower level of plasma tryptophan concentrations [33]. Therefore, TRS may be a distinct and homogenous subgroup of schizophrenia.

To identify the genetic variants susceptible for schizophrenia, this study performed the first GWAS focusing on TRS in a Han-Chinese population. We identified several novel genetic loci which were not associated with schizophrenia. Our findings may pave a new way to elucidate the underlying molecular mechanism of schizophrenia and to improve the treatment for TRS.

## Results

### Demographic information

Demographic data from 522 TRS patients and 806 controls is listed in Table 1. The mean age was 44.12±9.06 years in cases and 67.64±9.36 years in controls. The male made up approximately 55% in cases and 48% in controls. Of these patients, 121 patients (23.2%) had a family history of psychiatric disorders; 264 patients (50.6%) displayed psychotic symptoms before age 20; 289 patients (55.4%) had shown violent or homicidal behavior; and 197 patients (37.7%) had attempted suicide. All of the patients showed persistence of their illness for more than 5 years, with a persistent CGI-S score of 4 or higher under antipsychotic treatments.

**Table 1:** Demographic and clinical characteristics for TRS.

Characteristics	Patients with TRS (N = 522)	Controls (N = 806)
Male (%)	289 (55.4%)	383 (47.5%)
Age- years	44.12	67.64
Body-mass index#	24.74	24.25
smoking - no. (%)	225 (43.1%)	270 (33.5%)
regular drinker - no. (%)	44 (8.4%)	90 (11.2%)
Family history of psychiatric disease	121 (23.2%)	33 (4.09%)
onset before 20 yr	264 (50.6%)	-
violent or homicidal - no. (%)	289 (55.4%)	-
suicide attempt - no. (%)	197 (37.7%)	-
Recruitment for TRS - no. (%)		
two trials of standard antipsychotic treatments	90 (17.24)	-
clozapine	432 (82.76)	-
Severity of TRS : scale of CGI-S∧		
1. normal	0	-
2. borderline mentally ill	0	-
3. mildly ill	0	-
4. moderately ill	129 (24.7%)	-
5. markedly ill	265 (50.8%)	-
6. severely ill	115 (22.0%)	-
7. extremely ill	13 (2.5%)	-

# means the body-mass index is the weight in kilograms divided by the square of the height in meters.

∧ means more than 5 years of persistence of illness without period of good social or occupational functioning assayed by the severity of illness subscale of clinical global impression (CGI-S).

### Data quality

The average call rate was 99.8±0.3% for each subject genotyped in this study. Gender determined from the GWAS result for all the subjects were consistent with recorded data. 694,436 (79.99%) of the 868,114 SNP in the autosomes passed the quality control filter and had an average call rate of 99.8±0.4% (Supplementary Table S1). The results of principal component analysis showed no significance for population stratification between TRS patients and controls, (*P*>0.05, and Fst statistics between populations <0.001) (Supplementary Figure S1). Furthermore, genomic control with a variance inflation factor λ = 1.042 (trend test), estimated posterior to the regular GWAS, also indicated no substantial population stratification. These SNPs were then taken for further GWAS analysis.

### Association analysis

**Figure 1:**
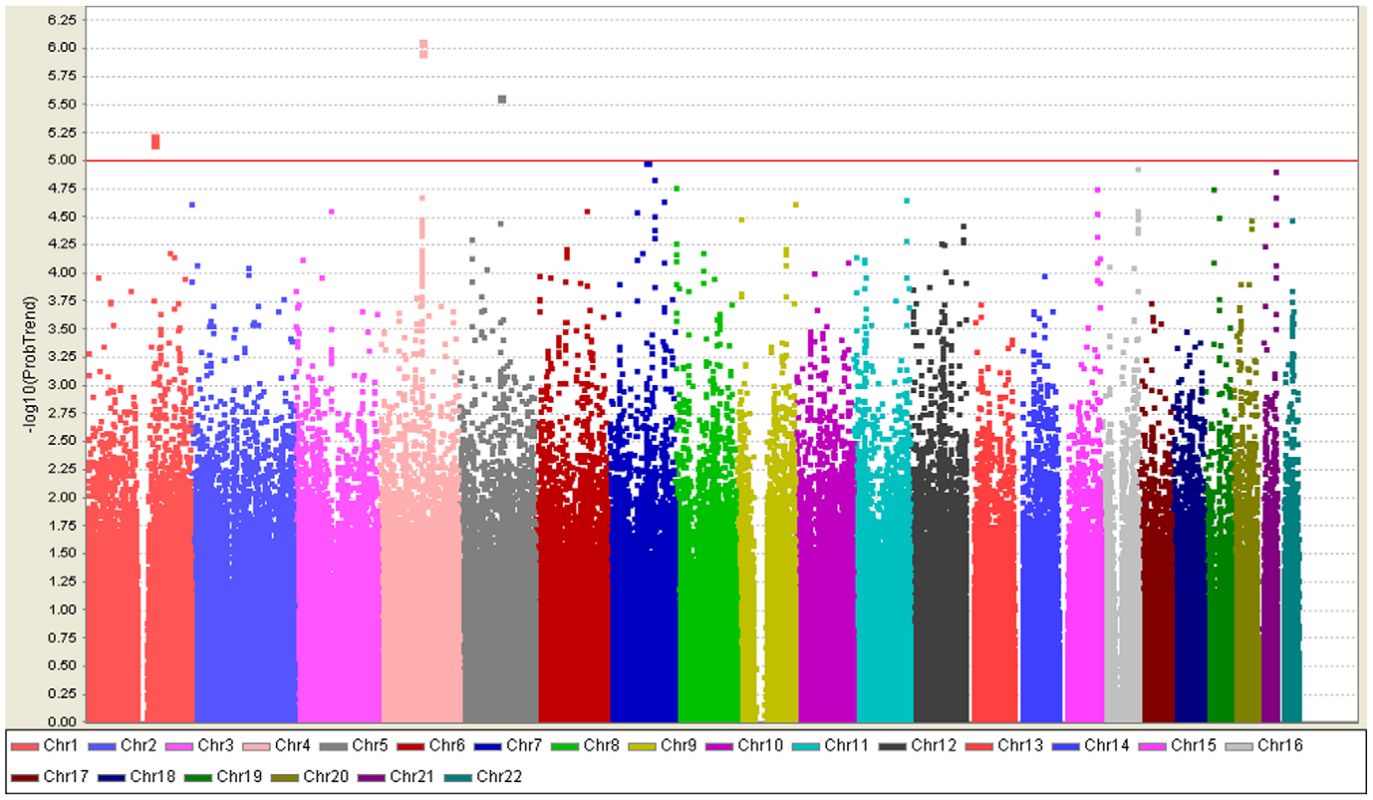
Graphical summary of genome-wide association analysis for TRS in a Han Chinese population. Results (−log_10_ *P*) are shown in chromosomal order for 694,436 SNPs which were tested in 522 cases and 806 controls by using Affymetrix SNP 6.0 Array. The horizontal line indicates a *P*-value of 10^−5^.

Data analysis was first performed for the 522 TRS patients and 806 controls (Figure 1). Preliminary results revealed 19 SNPs with suggestive significant associations with TRS (Supplementary Table S2, 10^−8^<*P*<10^−5^). Fourteen markers were retained after cross platform validation with the Sequenom platform and showed a concordance rate of over 98% (Supplementary Table S2).

**Figure 2:**
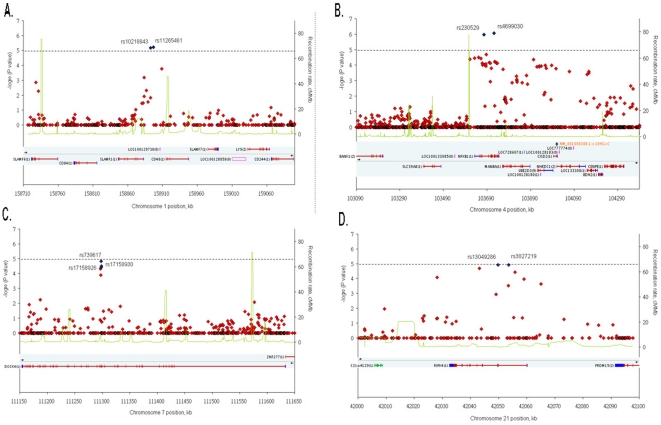
Refined regional association plots. For each plot of the four clusters ((A) *SLAMF1*, (B) *NFKB1*, (C) *DOCK4*, and (D) *RIPK4*), the −log_10_ *P* values for the trend test from Affymetrix SNP 6.0 Array in 522 cases and 806 controls are plotted as red diamond according to their genomic position (NCBI Build 36.3). The SNPs with the strongest signal are represented as blue diamonds. The recombination rates (right y-axis) based on the Chinese HapMap population is plotted as green lines to reflect the local LD structure around the SNPs. The dashed horizontal line indicates a *P*-value of 10^−5^.

Four major clusters with more than one SNPs located within 500 kb of each other were identified from the 14 validated SNPs (Table 2, Figure 2). The first locus, comprising rs10218843 (*P* = 6.73×10^−6^) and rs11265461 (*P* = 5.90×10^−6^), and is located approximately 10 kb downstream of signaling lymphocytic activation molecule family member 1 (SLAMF1) on chromosome 1; the second locus contains two SNPs, rs4699030 (*P* = 8.41×10^−7^) and rs230529 (*P* = 1.07×10^−6^), is located in the introns of nuclear factor of kappa light polypeptide gene enhancer in B-cells 1 (NFKB1) on chromosome 4; three SNPs, rs739617 (*P* = 1.46×10^−5^), rs17158926 (*P* = 3.99×10^−5^) and rs17158930 (*P* = 3.08×10^−5^) are clustered in the introns of dedicator of cytokinesis 4 (*DOCK4*) on chromosome 7; and the last locus which consists of two SNPs, rs13049286 (*P* = 1.23×10^−5^) and rs3827219 (*P* = 1.23×10^−5^), is located in receptor-interacting serine/threonine-protein kinase 4 (*RIPK4*) on chromosome 21. These loci are located in the regions with high LD (except for chromosome 21) (Supplementary Figure S2). Multipoint/Haplotype analysis also showed that these clusters were associated with TRS, the cluster on chromosome 4 had the highest *P* value with global score *P* = 2×10^−5^ (Supplementary Table S3).

**Table 2:** Results of GWAS for TRS in Han population.

ch	SNP	position	Allele	RA	RAF in control	RAF in case	F difference	*P*_trend_	OR (95% CI)	*P*_joint_	OR_joint_ (95% CI)	closest gene	Distance to gene (bp)
1	rs10218843	158892685	CT	C	0.407	0.495	0.088	6.73×10^−6^	1.43 (1.22–1.67)	3.04×10^−7^	1.45 (1.26–1.66)	*SLAMF1*	8980
1	rs11265461	158896767	CT	C	0.411	0.500	0.089	5.90×10^−6^	1.43 (1.22–1.68)	1.94×10^−7^	1.45 (1.26–1.67)	*SLAMF1*	13062
4	rs230529	103676448	CT	T	0.472	0.570	0.097	1.07×10^−6^	1.48 (1.27–1.73)	1.74×10^−7^	1.45 (1.26–1.66)	*NFKB1*	0
4	rs4699030	103722862	CG	C	0.470	0.568	0.098	8.41×10^−7^	1.48 (1.27–1.73)	1.94×10^−6^	1.40 (1.22–1.61)	*NFKB1*	0
5	rs461409	97957866	AG	G	0.793	0.864	0.071	2.63×10^−6^	1.65 (1.33–2.05)	4.50×10^−4^	1.39 (1.15–1.66)	*RGMB*	−175034
7	rs12533497	91495608	CT	T	0.071	0.122	0.051	1.04×10^−5^	1.81 (1.39–2.36)	1.69×10^−4^	1.60 (1.25–2.05)	*AKAP9*	0
7	rs739617	111298102	AG	A	0.130	0.191	0.061	1.46×10^−5^	1.58 (1.28–1.95)	3.87×10^−5^	1.50 (1.23–1.82)	*DOCK4*	0
7	rs17158926	111298199	AT	A	0.135	0.193	0.059	3.99×10^−5^	1.54 (1.25–1.90)	5.08×10^−4^	1.40 (1.16–1.70)	*DOCK4*	0
7	rs17158930	111298374	AG	G	0.134	0.193	0.059	3.08×10^−5^	1.55 (1.26–1.91)	3.98×10^−4^	1.41 (1.17–1.71)	*DOCK4*	0
8	rs9314462	2501291	CT	C	0.199	0.266	0.066	5.30×10^−5^	1.45 (1.21–1.75)	5.35×10^−4^	1.34 (1.13–1.59)	*CSMD1*	−278992
16	rs9646303	86019470	CT	C	0.409	0.496	0.087	1.15×10^−5^	1.42 (1.22–1.67)	3.33×10^−4^	1.30 (1.13–1.49)	*ZCCHC14*	0
19	rs11673496	22581270	AG	G	0.732	0.807	0.075	1.75×10^−5^	1.53 (1.27–1.85)	2.24×10^−4^	1.37 (1.16–1.61)	*ZNF492*	−60996
21	rs13049286	42049868	AC	C	0.014	0.041	0.027	1.23×10^−5^	3.08 (1.83–5.18)	3.05×10^−5^	2.78 (1.70–4.56)	*RIPK4*	0
21	rs3827219	42053555	AG	A	0.014	0.042	0.028	1.23×10^−5^	3.02 (1.81–5.03)	1.66×10^−5^	2.80 (1.73–4.55)	*RIPK4*	0

SNP position were indexed to the forward of NCBI Build 36.3.

ch: chromosome.

RA: Risk allele, the allele with higher frequency in schizophrenia as compared with controls;

RAF: risk allele frequency.

F: frequency.

*P*_trend_: P values obtained from the initial GWA analysis on 522 cases and 806 controls.

*P*_joint_: P values calculated from joint analysis on 804 cases and 806 controls.

OR, odds ratio for risk allele.

In addition to the above SNPs in clusters, five other SNPs also showed suggestive significant association with TRS. These SNPs are: rs461409 (*P* = 2.63×10^−6^) which is located 175 kb downstream of RGM domain family, member B (*RGMB*) on chromosome 5; rs123533497 (*P* = 1.04×10^−5^) in intron 14 of A-kinase anchor protein 9 (*AKAP9*) on chromosome 7; rs9314462 (*P* = 5.30×10^−5^) in the downstream of CUB and sushi domain-containing protein 1 (*CSMD1*) on chromosome 8; rs9646303 (*P* = 1.15×10^−5^) in intron 3 of zinc finger, CCHC domain containing 14 (*ZCCHC14*) on chromosome 16; and rs11673496 (*P* = 1.75×10^−5^), 60 kb upstream of zinc finger protein 492 (*ZNF492*) on chromosome 19 (Table 2, Supplementary Figure S3).

Except for rs13049286 and rs3827219 with odds ratio (OR) of approximately 3, all other SNPs identified in this study showed modest effects with OR between 1.06–1.81 (Table 2).

### Joint Analysis in with additional TRS patients

The 14 SNPs showing suggestive significance were then genotyped in an independent cohort of 273 TRS patients. An average call rate of 99.37±0.21% was achieved for each subject. Joint analysis was then carried out in the 795 cases and 806 controls. Of the 14 SNPs showing suggestive association in the initial analysis, 7SNPs remain suggestively associated with TRS after joint analysis (Table 2). These SNPs are: rs10218843 (*P*_joint_ = 3.04×10^−7^) and rs11265461 (*P*_joint_ = 1.94×10^−7^), which both are located in SLAMF1; rs230529 (*P*_joint_ = 1.47×10^−7^) and rs4699030 (*P*_joint_ = 1.94×10^−6^) are located in NFKB1; rs739617 (*P*_joint_ = 3.87×10^−5^) is in DOCK4; rs13049268 (*P*_joint_ = 3.05×10^−5^) and rs3827219 (*P*_joint_ = 1.66×10^−5^) which are located in RIPK4.

### Testing in schizophrenic patients

The top SNPs showing suggestive significance were then tested in an independent cohort of 1982 schizophrenic patients whose responses to antipsychotic treatments were not determined and additional 2000 controls. An average call rate of 99.37±0.21% was achieved for each subject. However, none of these SNPs were significantly associated with this group of patients (Supplementary Table S4), suggesting that these SNPs were specifically associated with TRS and not a broad phenotype of schizophrenia.

### Re-sequencing of *NFKB1*

**Figure 3:**
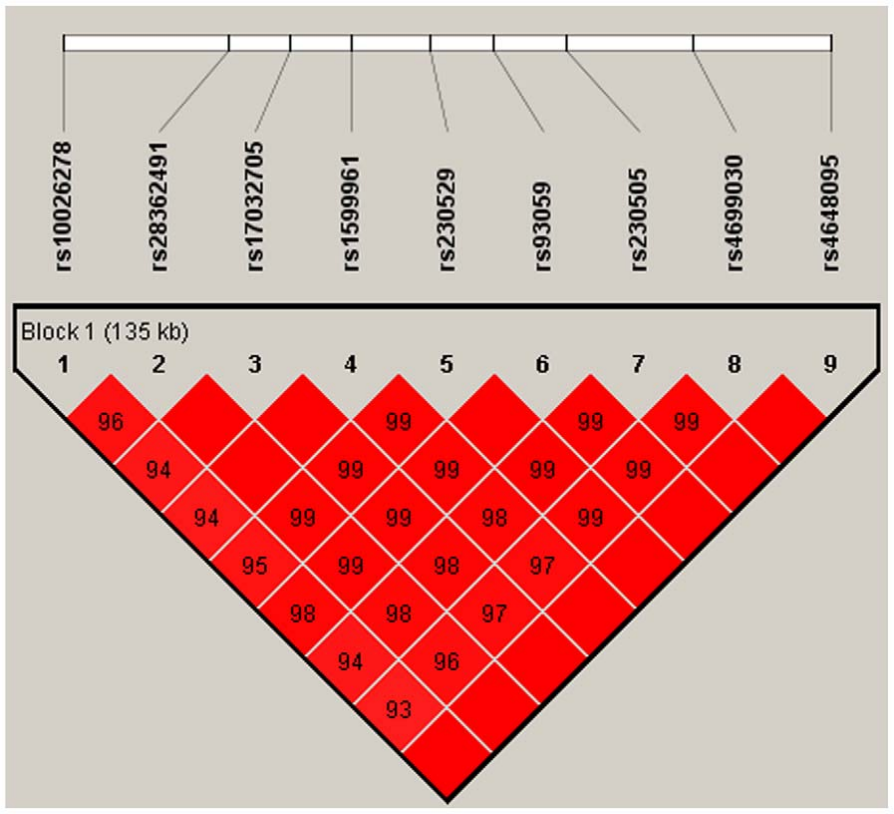
LD plot of rs28362491 (-94delATTG). rs28362491 shows linkage disequilibrium with the Affymetrix SNPs. Both rs230529 and rs4699030 have the lowest *P* value in this study.

Because the lowest *P* values in both single and multi-point analysis were observed for the SNPs located on *NFKB1* on chromosome 4, we next aimed to identify variants with functional consequence in *NFKB1*. Re-sequencing was performed on the exons, intron-exon boundaries, and a 2-kb region covering the promoter of *NFKB1* in a discovery cohort of 94 TRS patients and 94 controls. Twenty-three genetic polymorphisms including 11 novel variants and 2 non-synonymous changes (R231L and R534H) were identified in *NFKB1* (Table 3). The rs28362491 SNP with an ATTG deletion in the promoter region of *NFKB1* (94delATTG) was reported to affect nuclear protein binding and gene transcription in colonic epithelial cells [34]. In a test with 520 TRS cases and 806 controls, rs28362491 was associated with TRS (*P* = 6.69×10^−5^). rs28362491 is in linkage disequilibrium with rs230529 and rs4699030 (r^2^ = 0.741 and 0.714, respectively) (Figure 3).

**Table 3:** Variants identified in NFKB1 by direct sequencing in 94 TRS cases and controls.

SNP	Region	Allele	Genotype			Effect	Risk Allele	RAF		F difference
				case (%)	control (%)			case	control	
rs28362491	P	ATTG/-	ins/ins∶ins/-∶-/-	24.4 ∶ 46.7 ∶ 28.9	31.7 ∶ 47.8 ∶ 20.5		del	0.522	0.444	0.078
rs11940017	P	T>C	TT∶TC∶CC	85.9 ∶ 14.1 ∶ 0	95.7 ∶ 4.3 ∶ 0		C	0.071	0.022	0.049
rs11944443	p	A>G	AA∶AG∶GG	85.9 ∶ 14.1 ∶ 0	95.7 ∶ 4.3 ∶ 0		G	0.071	0.022	0.049
rs41477752	I2	T>-	TT∶T/-∶-/-	87.0 ∶ 13.0 ∶ 0	95.6 ∶ 4.4 ∶ 0		del	0.065	0.022	0.043
IVS2-60 A>G	I2	A>G	AA∶AG∶GG	98.9 ∶ 1.1 ∶ 0	100.0 ∶ 0 ∶ 0		G	0.005	0	0.005
c.692 G>T	E8	G>T	GG∶GT∶TT	98.9 ∶ 1.1 ∶ 0	100.0 ∶ 0 ∶ 0	R231L	T	0.005	0	0.005
rs4648049	I12	C>T	CC∶CT∶TT	86.2 ∶ 13.8 ∶ 0	95.5 ∶ 4.5 ∶ 0		T	0.069	0.022	0.047
rs4648050	I12	T>C	TT∶TC∶CC	25.5 ∶ 36.2 ∶ 38.3	27.0 ∶ 49.4 ∶ 23.6		C	0.564	0.483	0.081
IVS12+21 C>T	I12	C>T	CC∶CT∶TT	98.9 ∶ 1.1 ∶ 0	100.0 ∶ 0 ∶ 0		T	0.005	0	0.005
rs1020760	I11	G>C	GG∶GC∶CC	42.1 ∶ 40.0 ∶ 17.9	24.7 ∶ 50.6 ∶ 24.7		G	0.621	0.5	0.121
IVS11-56 T>C	I11	T>C	TT∶TC∶CC	97.9 ∶ 2.1 ∶ 0	98.9 ∶ 1.1 ∶ 0		C	0.011	0.006	0.005
c.1116 G>A	E12	G>A	GG∶GA∶AA	98.9 ∶ 1.1 ∶ 0	100.0 ∶ 0 ∶ 0	S372S	A	0.005	0	0.005
IVS13+196 T>G	I13	T>G	TT∶TG∶GG	98.9 ∶ 1.1 ∶ 0	100.0 ∶ 0 ∶ 0		G	0.005	0	0.005
c.1601 G>A	E15	G>A	GG∶GA∶AA	98.9 ∶ 1.1 ∶ 0	100.0 ∶ 0 ∶ 0	R534H	A	0.005	0	0.005
IVS15+12 C>G	I15	C>G	CC∶CG∶GG	98.9 ∶ 1.1 ∶ 0	100.0 ∶ 0 ∶ 0		G	0.005	0	0.005
IVS15+40 G>A	I15	G>A	GG∶GA∶AA	98.9 ∶ 1.1 ∶ 0	100.0 ∶ 0 ∶ 0		A	0.005	0	0.005
rs4648095	I17	T>C	TT∶TC∶CC	81.1 ∶ 18.9 ∶ 0	93.6 ∶ 9.6 ∶ 0		C	0.095	0.032	0.063
rs4648110	I22	T>A	TT∶TA∶AA	92.6 ∶ 7.4 ∶ 0	87.1 ∶ 12.9 ∶ 0		T	0.963	0.935	0.028
rs4648117	I22	C>T	CC∶CT∶TT	80.6 ∶ 19.4 ∶ 0	94.3 ∶ 5.7 ∶ 0		T	0.097	0.028	0.069
IVS22-23 C>T	I22	C>T	CC∶CT∶TT	97.8 ∶ 2.2 ∶ 0	100.0 ∶ 0 ∶ 0		T	0.011	0	0.011
rs3817685	I22	G>C	GG∶GC∶CC	32.6 ∶ 41.3 ∶ 26.1	20.5 ∶52.3 ∶ 27.3		G	0.533	0.466	0.067
rs35795162	I23	-/A	-/-∶-/A∶AA	77.4 ∶ 20.4 ∶ 2.2	90.9 ∶ 9.1 ∶ 0		A	0.124	0.045	0.079
IVS23-44 G>A	I23	G>A	GG∶GA∶AA	98.9 ∶ 1.1 ∶ 0	100.0 ∶ 0 ∶ 0		A	0.005	0	0.005

Risk allele means the allele with higher frequency in schizophrenia as compared with controls;

RAF: risk allele frequency.

P: promoter, I : intron.

### Functional analysis of rs28362491

**Figure 4:**
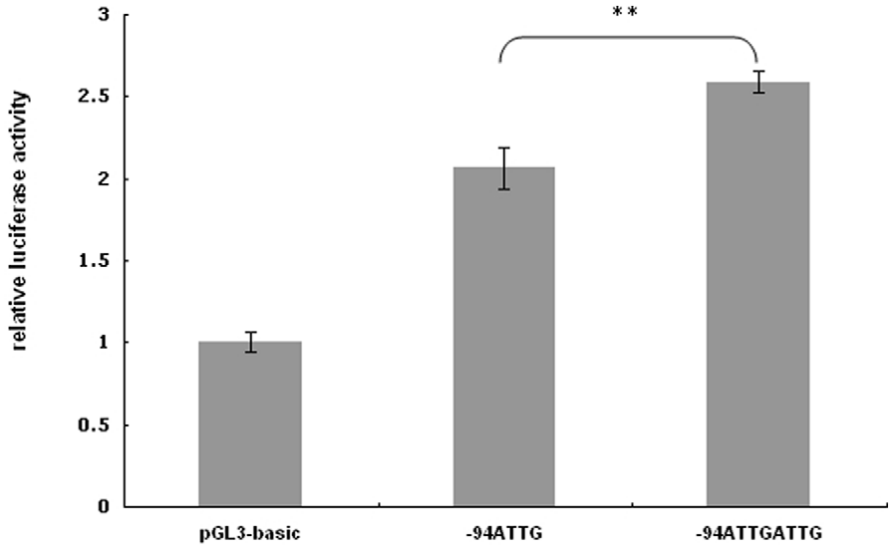
Reporter assay of rs28362491 (-94ins/delATTG) of *NFKB1* in SH-SY5Y cells. pGL3 luciferase reporter contained either the -94ATTGATTG (W) or the -94ATTG (D) allele at the promoter region of the *NFKB1*. Values represented the average of three experiments and the error bars represented the standard deviation. pGL3-basic was used as control without any promoter sequence inserted. ** *P*<0.01.

Since rs28362491 has been reported to affect nuclear protein binding and gene transcription in colonic epithelial cells, it is possible that the deletion also alters the efficiency of transcription in neuronal cells. The promoter assay showed that the construct containing the -94delATTG promoter displayed significantly reduced luciferase activity (2.07±0.13) as compared with the wild type construct (2.59±0.07) (*P* = 0.003) (Figure 4).

## Discussion

This study presented the data of the first GWAS on TRS in a Han-Chinese population. Therefore, the use of genomic control did not substantially change the results of this GWAS (Supplementary Table S5). The sample size is comparatively smaller than in previous GWAS for schizophrenia due to the smaller number of TRS patients available Since schizophrenia is an etiologically heterogeneous disorder, narrowing schizophrenia down to TRS may represent a more discrete and genetically homogeneous group to identify genes involved in the etiology of schizophrenia.

Among the genetic loci with suggestive significance identified by this study, three regions with more than one significant SNP in each region stood out after joint analysis. rs4699030 and rs230529 are located in the introns of *NFKB1*. This gene encodes for two functional different proteins [35], one for the 105 kD (p105) protein, and the other for a 50 kD (p50) protein. P105 contains seven copies of ankyrin-like sequence in the carboxyl terminal region which is similar to those in I-kappa B kinase (IκB), therefore, p105 may also have functions similar to IκB. P105 could associate with either c-Rel or RelA in the cytoplasm to inhibit Rel protein-specific transcription [36], [37]. P50 is one subunit of NF-kappa B with repression activity. NF-kappa B is well distributed and a highly conserved dimeric transcriptional factor which regulates more than 200 genes [38]. Different dimeric combinations of NF-kappa B are found in different tissues and respond differently to regulate gene expressions. P50 assembles either with other NF-kappa B subunits, such as RelA, RelB, c-Rel, or with itself as a homodimer to repress NF-kappa B–dependent gene transcriptions [39]. The heterodimer of p50 and RelA subunit is the most abundant form of NF-kappa B [40].

Both rs10218843 and rs11265461 are located near *SLAMF1* (also known as CD150), which is a signaling lymphocyte activation molecule and a member of the CD2 family belonging to the immunoglobulin superfamily of receptors. SLAM is a co-stimulatory molecule involving T cell activation and also as a receptor for measles virus [41], a bacterial sensor [42], and responsible for the NKT lineage development [43].The activation of SLAM has been shown to associate with numerous distinct downstream activities, including augmenting T cell mediated cytotoxicity through a sequence of signaling transduction, including NF-kappa B activation, Stat1 phosphorylation and T-bet induction [44]; increasing recruitment of protein kinase C (PKC)-theta and the activation of NF-kappa B p50, both of which are involved in enhancing T helper 2 cytokines production and natural killer T cell development [45], [46].

Two SNPs, rs13049286 and rs3827219 are both located in *RIPK4*. The expression of RIPK4 is well distributed, including the brain, and found to interact with PKC-delta [47]. This gene is important in sensing cellular stress, such as infection, inflammation, cellular differentiation programs and DNA damage. It also mediates downstream signaling, in particular the activation of NF-kappa B and the induction of apoptosis [48].

The identification of *NFKB1*, *RIPK4*, and *SLAMF1* in this study suggests that the NF-kappa B pathway plays an important role in the pathogenesis of TRS. NF-kappa is also found to be related with schizophrenia since the genetic variants in RELA gene, which encoded the protein RelA as one subunit of NF-kappa B, were reported to be associated with schizophrenia and the patients startle responses in a Japanese population [49]. NF-kappa B is a key transcriptional factor in the regulation of the expression of many inflammatory factors, such as cytokines, chemokines, and adhesion molecules [50]. Several studies have demonstrated that schizophrenic patients' cerebrospinal fluid and plasma had abnormal levels of cytokines [51], [52], [53], the aberrations were especially more pronounced in TRS [54], [55], [56]. Song et al. found the elevated level of cytokine in first-episode schizophrenic patients was associated with the activation of NF-kappa B [53]. Thus, these suggest abnormal inflammatory response could lead to TRS. One common variant rs28362691 (-94ins/delATTG) identified from resequencing *NFKB1* was found to associate with TRS. This SNP is located in 19 base pairs upstream of a functional κB binding site [57]. The promoter assay showed that the *NFKB1* promoter with the -94delATTG allele had a lower promoter activity in SH-SY5Y cells in comparison with the -94insATTG allele. This implies that the -94delATTG allele may result in lower expression of *NFKB1*. Changes in *NFKB1* expression could alter the level of p105 and induce divergent dimeric combinations of NF-kappa B, which might cause disturbances in cytokine regulations, and lead to a failure of antipsychotic treatment. However, the association between the abnormal levels of cytokines and NF-kappa B in patients with TRS remains to be established. Two novel non-synonymous polymorphisms (R231L and R534H) were also identified from resequencing. However, these polymorphisms have extremely low frequency in the Han population and their effects on *NFKB1* function remain to be elucidated.

Other genetic loci identified in association with TRS in this study suggests genes involved in neuronal development (*DOCK4* [58]). A recent study conducted in the Jewish population also identified a SNP (rs2074127) in DOCK4 associated with schizophrenia [59] however, this SNP is not in LD with the DOCK4 SNPs reported in this study (Supplementary Figure S4). Its role in the development of TRS remains to be elucidated.

We also compared our results with previous genetic studies showing associations with TRS (such as variants in *CYP3A4* [60], *CYP3A5* [60], *DRD3* [60], *HTR2A* [61], *HTR3A* [62]), none of the variants or their nearby SNPs had a *P* value lower than 10^−5^ in this study. It implicated that these above genes involving in metabolic enzymes and receptors of antipsychotics were not associated with TRS. Furthermore, we also compared our data with previous GWAS on schizophrenia. We examined the *P* values of the loci previously reported to be associated with schizophrenia in our data. We also checked the vicinity (200 kb) of the reported loci in our data. Only rs1602565 on chromosome 11 showed nominal association (*P* = 3.17×10^−4^) in this study (Supplementary Table S6 and Supplementary Figure S5). These data suggested that the genetic loci identified in this study were specifically associated with TRS.

None of the loci identified in this study reached genome-wide significance, this could due to tour sample size lack the statistical power to detect common variants of susceptibility. However, we focused on TRS within schizophrenia, which may represent a more homogeneous group. An independent TRS group was also not available for replication study. Future replication studies in additional population of TRS are required.

In conclusion, we report the first GWAS on TRS in the Han Chinese population. Our data suggest that the NF-kappa B pathway may play an important role in the pathogenesis of TRS. We have also provided the functional effect of the -94delATTG allele showing the possible mechanism of *NFKB1* in TRS. Further studies are required to confirm the association of the risk alleles identified in this study across different populations, to identify the causative genetic variants, and to elucidate the underlying molecular mechanisms, which may help to improve treatments for refractory schizophrenia.

## Materials and Methods

### Subjects

This study was approved by the Institute Review Board of Taipei Veterans General Hospital Kaohsiung, Kai-Suan Psychiatric Hospital, Jianan Mental Hospital, Bali Psychiatric Center, Tsyr-Huey Mental Hospital, Yuli Veterans Hospital, National Taiwan University Hospital and Academia Sinica. Written informed consent was obtained from all the study participants.

A total of 522 unrelated patients with TRS, including 289 males (55.4%) and 233 females (44.6%), were recruited from Yuli Veterans Hospital, Taipei Veterans General Hospital, Kaohsiung Kai-Suan Psychiatric Hospital, Tsyr-Huey Mental Hospital, Jianan Mental Hospital, and Bali Psychiatric Center. DNA samples from additional 273 TRS patients were obtained from National Taiwan University Hospital and were used in joint analysis. In addition, DNA samples from 1982 schizophrenic patients were obtained from Yuli Veterans Hospital, Taipei Veterans General Hospital, and National Health Research Institutes. However, the responses to antipsychotic treatments for 1982 schizophrenic patients were not determined.

All patients were diagnosed according to the criteria of DSM-IV for schizophrenia. TRS was defined using a modified Conley and Kelly's protocol [28]. Briefly, schizophrenic patients with the following criteria were identified as TRS: No improvement in clinical impression (defined as 3 or more in the global improvement subscale of clinical global impression (CGI-I)) after at least two six-week trials of antipsychotic therapy at a dose equal to or higher than the equivalent daily dose of 600 mg of chlorpromazine for typical antipsychotics, or for second-generation antipsychotics (risperidone: 6 mg/day; olanzapine: 20 mg/day; quetiapine: 800 mg/day; ziprasidone: 160 mg/day; amisulpride: 800 mg/day; zotepine: 300 mg/day), as well as for patients who were administered the last-line antipsychotic pharmacotherapy, clozapine (50–300 mg/day). All patients with TRS showed more than 5 years of persistent illness (defined as 4 or more in the severity of illness subscale of clinical global impression (CGI-S)). Informed consent was obtained from all participants. Only the Han-Chinese population, which accounts for 98% of the population in Taiwan, was recruited for this study.

The control (N = 2806) was randomly selected from the Han-Chinese Cell and Genome Bank in Taiwan described previously [63], in which more than 3,300 controls were collected and randomly selected through registry.

### Genotyping and Quality Control

Genomic DNA was isolated from peripheral blood using PUREGENE DNA purification system (Gentra Systems, Minneapolis, MN). Whole-genome scan was conducted using Affymetrix® Genome-wide Human SNP Array 6.0 (Affymetrix, Santa Clara, CA, USA) on 522 TRS patients and 806 controls and genotyping was performed by National Genotyping Center at Academia Sinica. Genotype calling was determined by Affymetrix Power Tool 1.10.2 (Affymetrix) using default parameters.

Quality control of genotype data was performed by examining several summary statistics. First, individual's gender was double checked by calculating the ratio of loci with heterozygous calls on the X chromosome; After calculating total successful call rate and the minor allele frequency (MAF) of cases and controls for each SNP, SNPs were excluded if one of the following conditions applied: (1) only one allele appeared in both cases and controls; (2) the total call rate was less than 98%; (3) the total MAF was less than 5% and the total call rate was less than 99%; (4) significant deviation from Hardy-Weinberg equilibrium in the control group (*P*<10^−4^).

### Population stratification

Detection of possible population stratification that might influence association analysis was carried out using principal component analysis (supplementary Methods S1, Supplementary Table S7) with genotype data for 100,000 SNPs located at equally spacing across the human genome. Plink (Supplementary Methods S2) was performed to examine if the subjects were related with each other. Variance inflation factor for genomic control was estimated based on all qualified SNPs (Supplementary Methods S3).

### Data Analysis

Genotyping data analysis was carried out by comparing the frequencies of allele and genotype between cases and controls using the following single-point methods: genotype, allele-type, trend test (Cochran-Armitage test), dominant, and recessive models. Empirical *P*-values were also obtained with 10^8^ simulations. SNPs with *P*-values less than α = 10^−8^, a cut-off for the multiple-comparison adjusted by Bonferroni correction, were considered to be significantly associated with the traits. SNPs with *P*-values between 10^−8^ and 10^−5^ were considered to have suggestive significant association. Quantile–quantile (Q–Q) plots were then used to examine *P*-value distributions (Supplementary Figure S6).

Multi-point/haplotype analysis was performed using the Haplotype Score Test [64] implemented in haplo.stat, a suite of S-PLUS/R routines for the analysis of indirectly measured haplotypes, for regions with more than two SNPs having genetic evidences (*P*-value<10^−5^). Regions were tested with independent 10^6^ simulations.

### Validation and joint analysis

Autosomal SNPs with *P*-value<10^−5^ from GWAS in 522 TRS cases and 806 controls were further validated using MALDI-TOF mass spectrometry (SEQUENOM MassARRAY, Sequenom, San Diego, CA, USA). The SNPs retained after cross-platform validation were then genotyped in the additional 273 TRS cases. Joint analysis was then carried out with all the 795 TRS cases and 806 control.

### Direct Sequencing

Selected candidate genes were re-sequenced in a discovery cohort consisted of 94 TRS patients and 94 controls. Exons, 200 bp of exon/intron junctions, and a 2–kb region covering the promoter of the selected genes were sequenced using Applied Biosystems 3730 (CA, USA). Contig assembly and SNP identification were determined using Sequencher 4.5 Demo (Gene Codes Cooperation, Ann Arbor, MI, USA). All PCR products were bi-directionally sequenced.

### Plasmid Construction for Luciferase Reporter Assay

To assay for the NFKB1 promoter activity, the NFKB1 promoter encompassing the -94ATTG polymorphism (from −1000 to −1) from patients with homozygous -94ATTGATTG (W) or -94ATTG (D) were first cloned into the pGEM-T Easy vector (Promega, Madison, WI, USA) with the forward primer: 5′-CCCGGGCCTGATTACTGATGTTTTAAAGCT-3′ and the reverse primer: 5′-CTCGAGTTCCTGGCTGGAAATTCCCACTGA-3′. Both the W and D fragments were then released from the pGEM-T Easy vector and subcloned into the upstream region of the firefly luciferase gene of the pGL3-basic vector (Promega). All constructs were subjected to sequencing to confirm the orientation and integrity.

### Transient Transfection and Luciferase Assay

A total of 1×10^5^ SH-SY5Y cells were seeded in each well of a 24-well plate and transfected with 450 ng of each reporter construct along with 50 ng of pRL-TK vector (Promega) containing the Renilla luciferase gene as an indicator for normalization of transfection efficiency. Transfections were performed by using FuGENE HD (Roche Applied Science, Indianapolis, IN, USA) according to manufacturer's instructions. Cells were incubated for 24 hours and analyzed for luciferase activity with the Dual-Luciferase Assay System (Promega). Firefly luminescence was normalized to Renilla luminescence and reported as relative luciferase activity. All experiments were performed in triplicate and repeated at least three times.

## Supporting Information

Figure S1 Principal component analysis (PCA) plot. The PCA plot shows the first two principal components, estimated by EIGENSTRAT (Price et al. Nature Genetics 38, 904–909 (2006)), which was based on genotype data from 100,000 SNPs with equally spacing across the human genome. No population stratification between the 502 TRS cases (CA, marked as blue circle) and 806 controls (CN, marked as pink cross) was detected (P>0.05, and Fst statistics between populations <0.001).
(DOCX)

Figure S2 LD blocks of the clusters showing suggestive significant association. LD (r^2^and D') blocks of the clusters on chromosome 1 (A), chromosome 4 (B), chromosome 7(C), and chromosome 21 (D), the validated SNPs with *P* value lower than 10^−5^ are marked in blue.
(DOCX)

Figure S3 Refined regional association plots for the five singleton SNPs with suggestive association. For each of the SNP (A) rs461409, (B) rs123533497, (C) rs9314462, (D) rs9646303, and (E) rs11673496, the −log_10_ *P* values for the trend test from Affymetrix SNP 6.0 Array in 522 cases and 806 controls are plotted as red diamond according to their genomic position (NCBI Build 36.3). The SNPs with the strongest signal are represented as blue diamonds. The recombination rates (right y- axis) based on the Chinese HapMap population is plotted as green lines to reflect the local LD structure around the significant SNPs. The dashed horizontal line indicates a *P*-value of 10^−5^.
(DOCX)

Figure S4 LD blocks of the DOCK4 SNPs.
(DOCX)

Figure S5 Comparisons to previous GWAS. For each of the (A) *PTBP2*, (B) *PLXNA2*, (C) *ZNF804A*, (D) *FXR1*, (E) MHC region/*SLC17A1/SLC17A3/BTN2A2/HIST1H2BJ/PRSS16/POM121L2/ZNF184/PGBD1*, (F) MHC region/*NOTCH4*/*HLA-DQA1*, (G) *RELN*, (H) *SMARCA2*, (I) *PLAA*, (J) *ANK3*, (K) Intergenic region on 11p14.1, (L) *NRGN*/I1 of *HEPACAM*, (M) Intergenic region on 16p13.2, (N) *ACSM1*, (O) *TCF4*, the −log_10_ *P*-values from primary scan are ploted as a function of genomic position (NCBI Build 36). The reported SNPs in previous GWAS are denoted by blue diamonds. Estimated recombination rates (right y axis) based on the Chinese HapMap population is plotted to reflect the local linkage disequilibrium structure around the significant SNPs. Gene annotations and number of transcripts were taken from NCBI.
(DOC)

Figure S6 Quantile-quantile (QQ) plots. QQ plot is shown for the trend test. *P*-values are based on the 694,436SNPs which passed quality filters from 522 cases and 806 controls. The upper and lower boundaries of the 95% confidence bands are represented by the blue lines.
(DOCX)

Table S1 Quality control of the genotyping results. Breakdown of the number (N) of SNPs and samples which passed the QC filter.
(DOCX)

Table S2 Concordance rates for the 19 SNPs with significant associations in the initial GWA analysis.
(DOCX)

Table S3 Multipoint/haplotype analysis of the clusters on chromosome 1 (A), chromosome 4 (B), and chromosome 7(C).
(DOCX)

Table S4 Testing TRS association results with schizophrenia.
(DOCX)

Table S5 SNPs showing suggestive significant associations adjusted using genomic control.
(DOCX)

Table S6 Previously reported loci and SNPs associated with schizophrenia.
(DOCX)

Table S7 Adjustment of the top SNPs for drinking and inclusion of 20 principal components as covariates in logistic regression.
(DOCX)

Methods S1 Principal component analysis using EIGENSTRAT.
(DOCX)

Methods S2 Analyses based on pair-wise identity-by-state (IBS) distance using Plink.
(DOCX)

Methods S3 Genomic Control.
(DOCX)
