# Supplementary material for: Genome-Wide Association Study of Treatment Refractory Schizophrenia in Han Chinese
Source: PLoS One. 2012 Mar 27;7(3):e33598. doi: 10.1371/journal.pone.0033598 (PMC3313922; doi:10.1371/journal.pone.0033598)
Supplement: Figure S1 — Principal component analysis (PCA) plot. The PCA plot shows the first two principal components, estimated by EIGENSTRAT (Price et al. Nature Genetics 38, 904–909 (2006)), which was based on genotype data from 100,000 SNPs with equally spacing across the human genome. No population stratification between the 502 TRS cases (CA, marked as blue circle) and 806 controls (CN, marked as pink cross) was detected (P>0.05, and Fst statistics between populations <0.001). (DOCX) [file pone.0033598.s001.docx]

**Supplementary Figure 1** Principal component analysis (PCA) plot.

The PCA plot shows the first two principal components, estimated by EIGENSTRAT (Price et al. *Nature Genetics* 38, 904-909 (2006)), which was based on genotype data from 100,000 SNPs with equally spacing across the human genome. No population stratification between the 502 TRS cases (CA, marked as blue circle) and 806 controls (CN, marked as pink cross) was detected (*P* > 0.05, and Fst statistics between populations < 0.001).

**
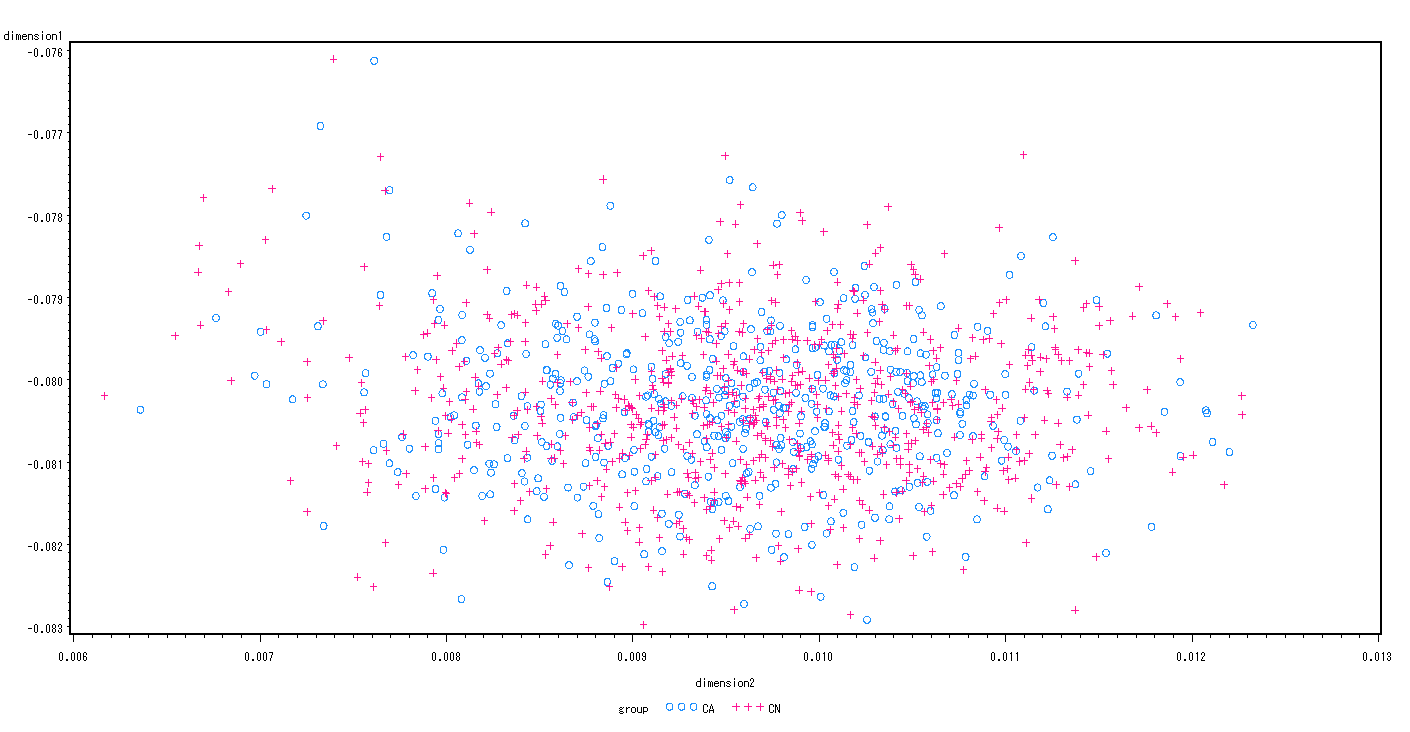
**
