# Supplementary material for: Genome-Wide Association Study of Treatment Refractory Schizophrenia in Han Chinese
Source: PLoS One. 2012 Mar 27;7(3):e33598. doi: 10.1371/journal.pone.0033598 (PMC3313922; doi:10.1371/journal.pone.0033598)
Supplement: Figure S2 — LD blocks of the clusters showing suggestive significant association. LD (r2and D') blocks of the clusters on chromosome 1 (A), chromosome 4 (B), chromosome 7(C), and chromosome 21 (D), the validated SNPs with P value lower than 10−5 are marked in blue. (DOCX) [file pone.0033598.s002.docx]

**Supplementary Figure 2** LD blocks of the clusters showing suggestive significant association.

LD (r^2^and D') blocks of the clusters on chromosome 1 (A), chromosome 4 (B), chromosome 7(C), and chromosome 21 (D), the validated SNPs with *P* value lower than 10^-5^ are marked in blue.

1. The cluster on chromosome 1

R^2^


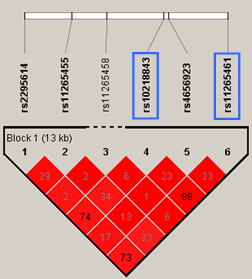


D'


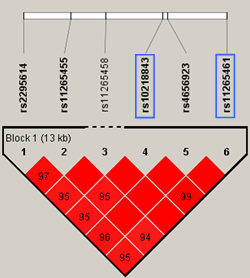


1. The cluster on chromosome 4

R^2^


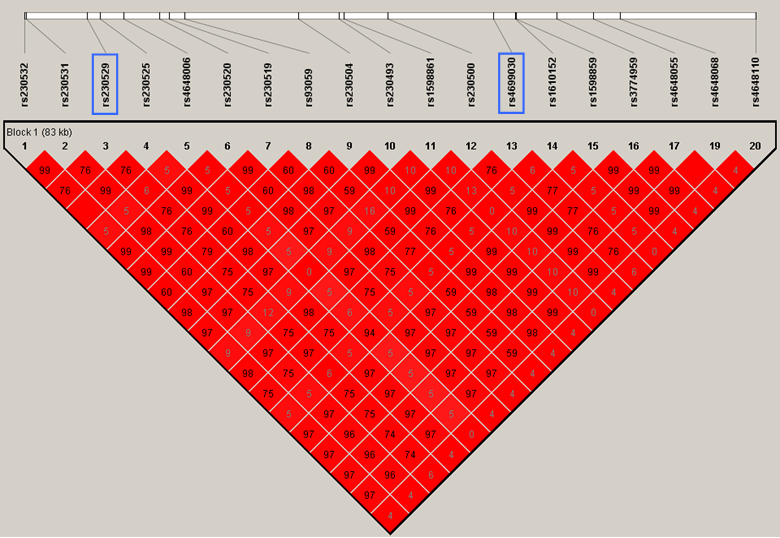


D'


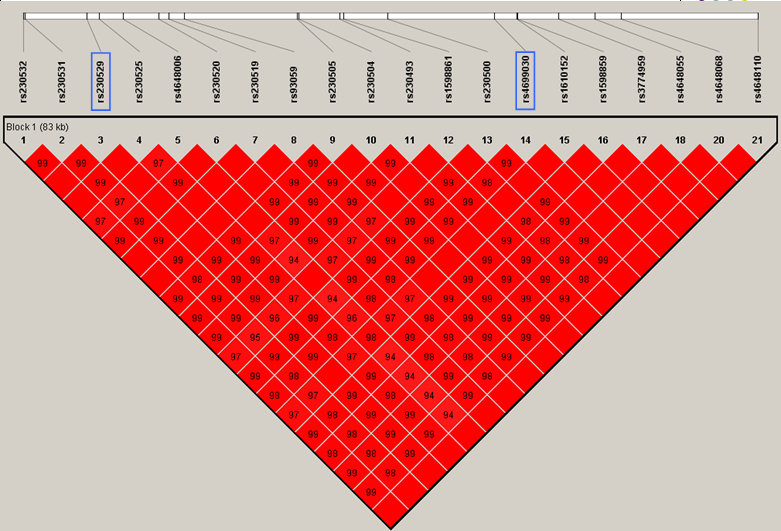


1. The cluster on chromosome 7

R^2^


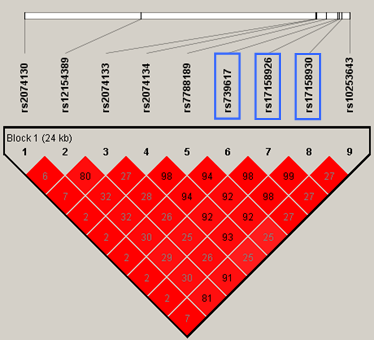


D'


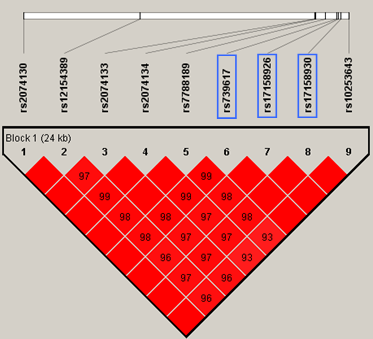


1. The cluster on chromosome 21

R^2^

**
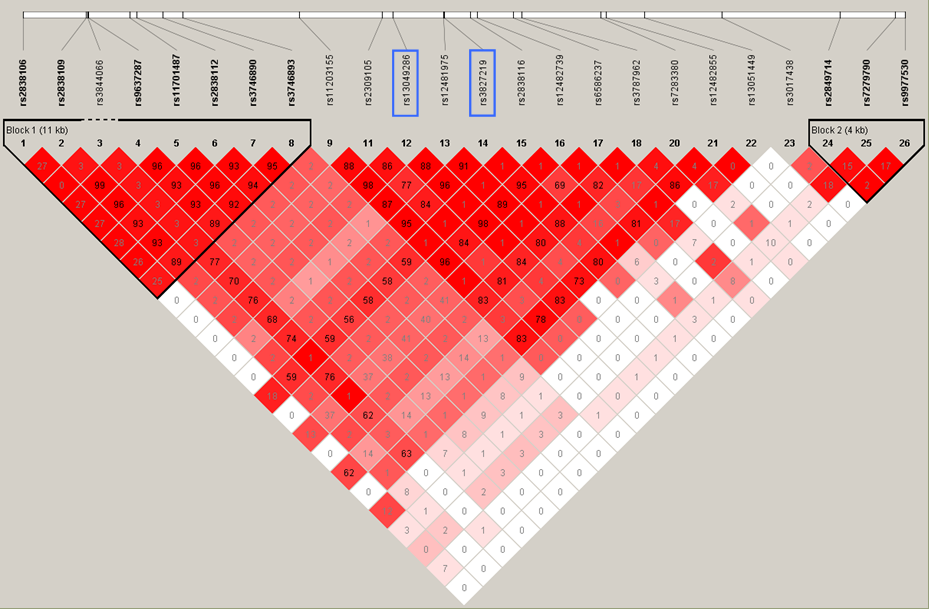
**

**D'**

**
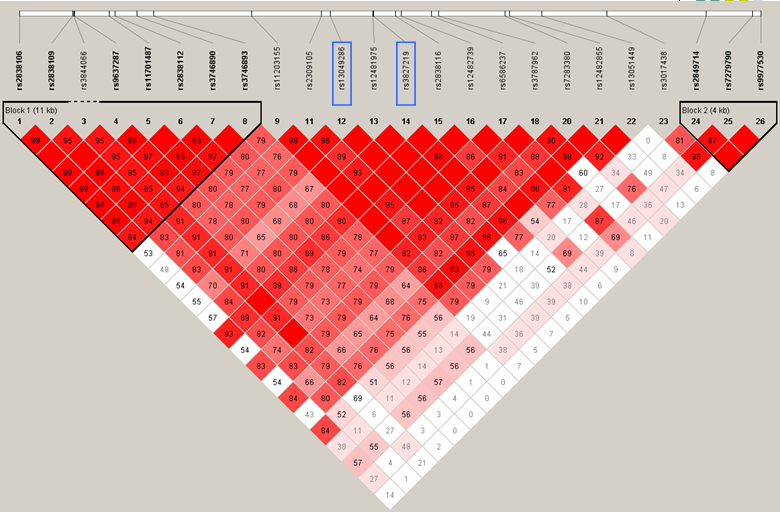
**
