# Supplementary material for: Genome-Wide Association Study of Treatment Refractory Schizophrenia in Han Chinese
Source: PLoS One. 2012 Mar 27;7(3):e33598. doi: 10.1371/journal.pone.0033598 (PMC3313922; doi:10.1371/journal.pone.0033598)

**Supplementary Figure 3** Refined regional association plots for the five singleton SNPs with suggestive association.

For each of the SNP (A) rs461409, (B) rs123533497, (C) rs9314462, (D) rs9646303, and (E) rs11673496, the -log_10_*P* values for the trend test from Affymetrix SNP 6.0 Array in 522 cases and 806 controls are plotted as red diamond according to their genomic position (NCBI Build 36.3). The SNPs with the strongest signal are represented as blue diamonds. The recombination rates (right y- axis) based on the Chinese HapMap population is plotted as green lines to reflect the local LD structure around the significant SNPs. The dashed horizontal line indicates a *P*-value of 10^−5^.

1. *Rs461409*

**
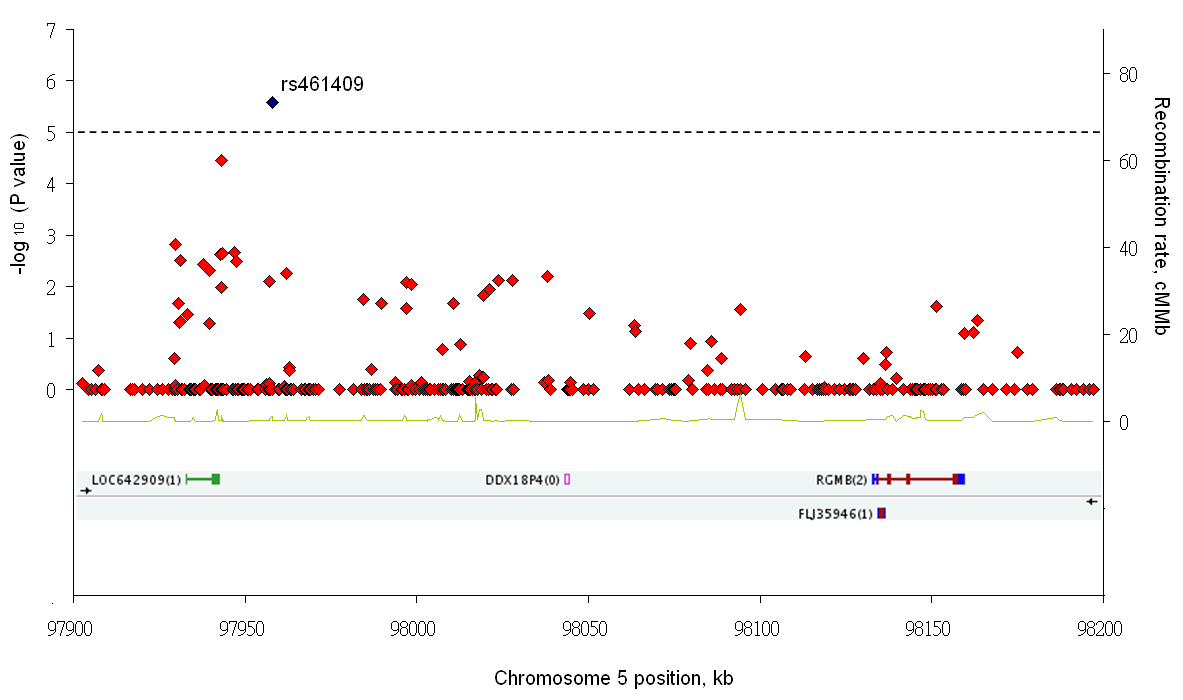
**

1. *rs12533497*

**
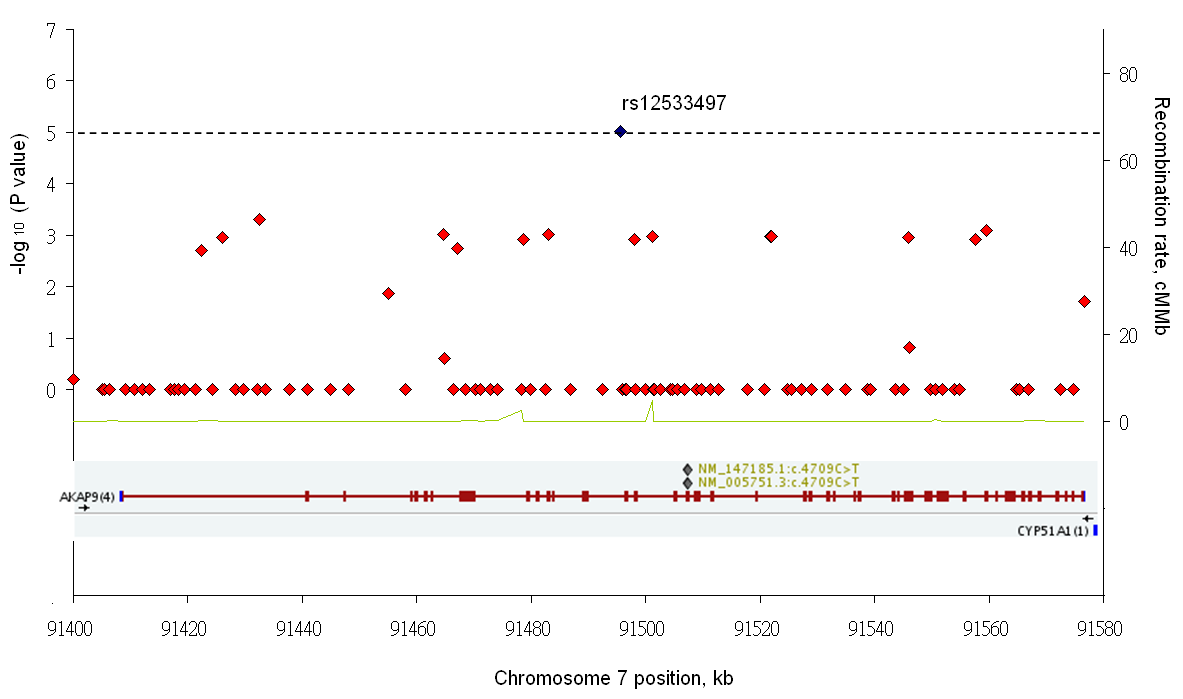
**

1. *rs9314462*

**
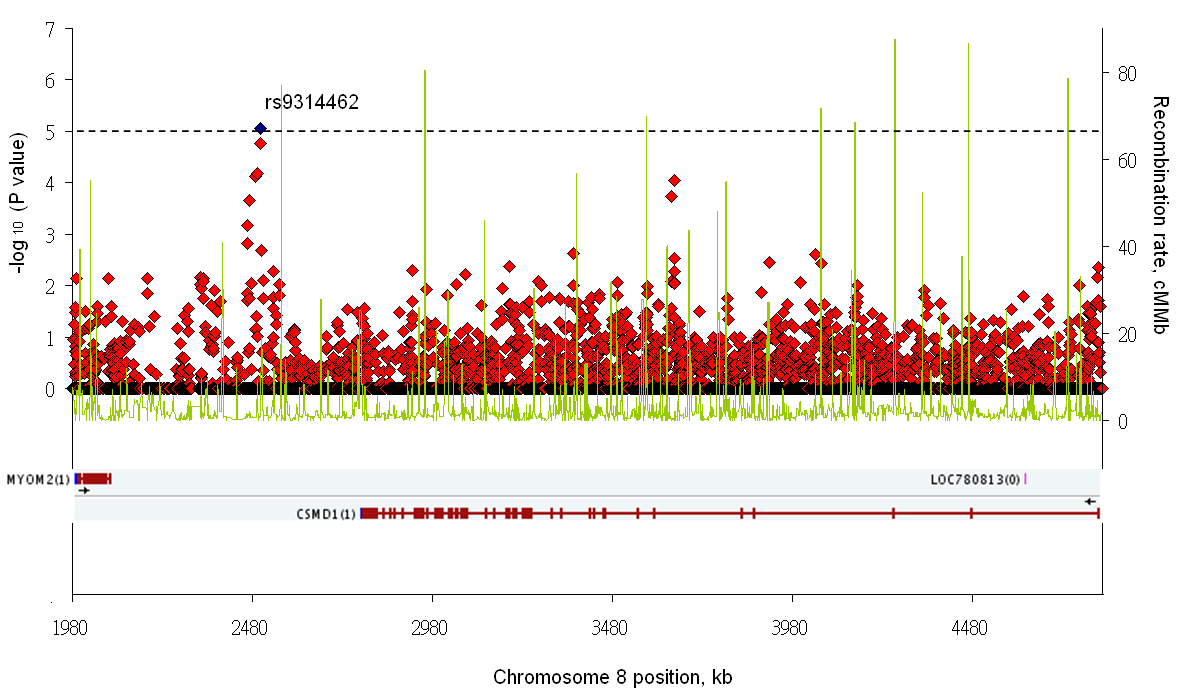
**

1. *rs9646303*


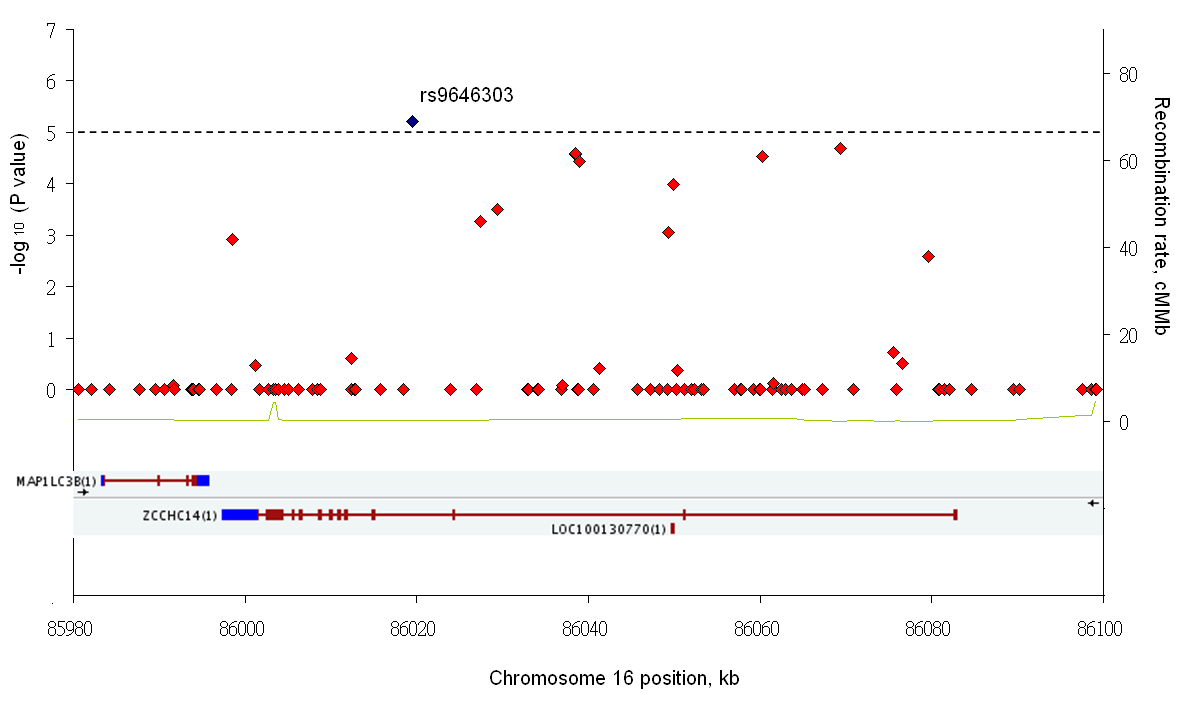


1. *rs11673496*


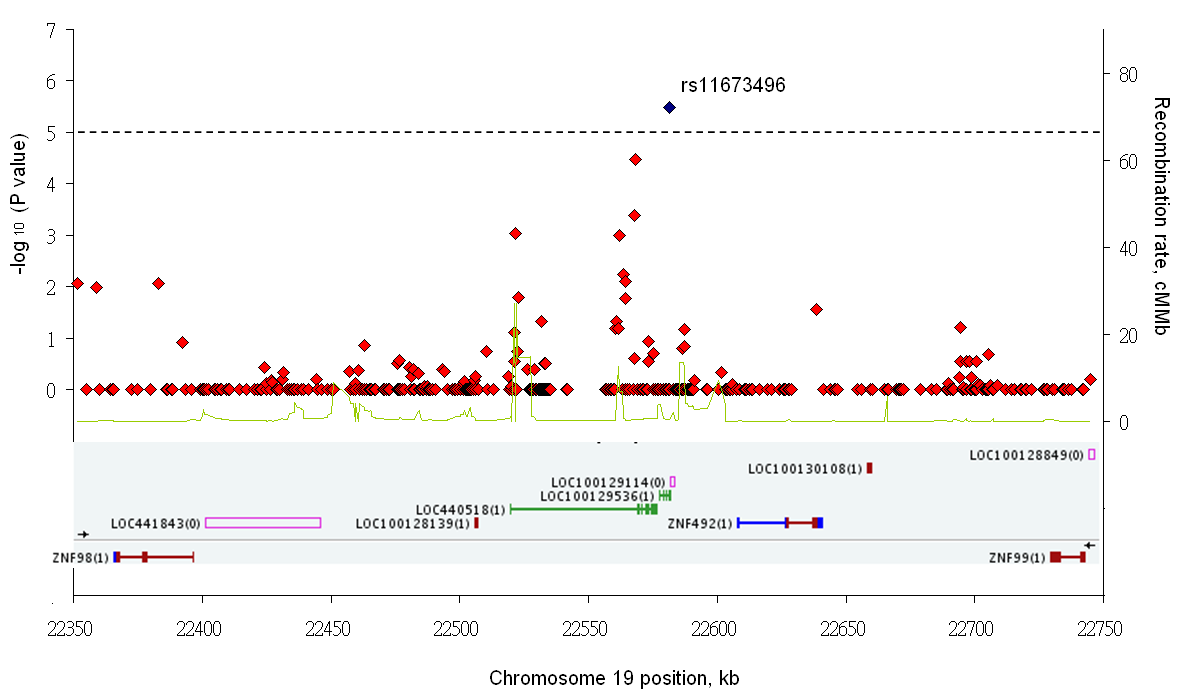

Supplement: Figure S3 — Refined regional association plots for the five singleton SNPs with suggestive association. For each of the SNP (A) rs461409, (B) rs123533497, (C) rs9314462, (D) rs9646303, and (E) rs11673496, the −log10 P values for the trend test from Affymetrix SNP 6.0 Array in 522 cases and 806 controls are plotted as red diamond according to their genomic position (NCBI Build 36.3). The SNPs with the strongest signal are represented as blue diamonds. The recombination rates (right y- axis) based on the Chinese HapMap population is plotted as green lines to reflect the local LD structure around the significant SNPs. The dashed horizontal line indicates a P-value of 10−5. (DOCX) [file pone.0033598.s003.docx]
