# Supplementary figures and images for: Genome-Wide Association Study of Treatment Refractory Schizophrenia in Han Chinese
Source: PLoS One. 2012 Mar 27;7(3):e33598. doi: 10.1371/journal.pone.0033598 (PMC3313922; doi:10.1371/journal.pone.0033598)

**Supplementary Figure 4** LD blocks of the DOCK4 SNPs

R2:


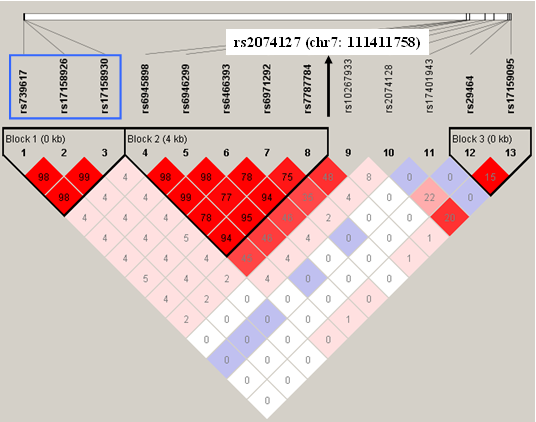


D':


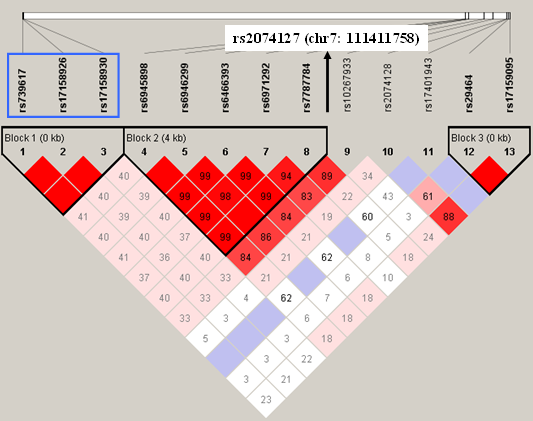

Supplement: Figure S4 — LD blocks of the DOCK4 SNPs. (DOCX) [file pone.0033598.s004.docx]
