# Supplementary material for: Genome-Wide Association Study of Treatment Refractory Schizophrenia in Han Chinese
Source: PLoS One. 2012 Mar 27;7(3):e33598. doi: 10.1371/journal.pone.0033598 (PMC3313922; doi:10.1371/journal.pone.0033598)

\

**Supplementary Figure 5** Comparisons to previous GWAS.

For each of the (A) *PTBP2*, (B) *PLXNA2*, (C) *ZNF804A*, (D) *FXR1*, (E) MHC region/ *SLC17A1/ SLC17A3/ BTN2A2/ HIST1H2BJ/ PRSS16/ POM121L2/ ZNF184/ PGBD1*, (F) MHC region/ *NOTCH4*/ *HLA-DQA1*, (G) *RELN*, (H) *SMARCA2*, (I) *PLAA*, (J) *ANK3*, (K) Intergenic region on 11p14.1, (L) *NRGN*/ I1 of *HEPACAM*, (M) Intergenic region on 16p13.2, (N) *ACSM1*, (O) *TCF4*, the –log_10_*P*-values from primary scan are ploted as a function of genomic position (NCBI Build 36). The reported SNPs in previous GWAS are denoted by blue diamonds. Estimated recombination rates (right y axis) based on the Chinese HapMap population is plotted to reflect the local linkage disequilibrium structure around the significant SNPs. Gene annotations and number of transcripts were taken from NCBI.

1. *PTBP2*


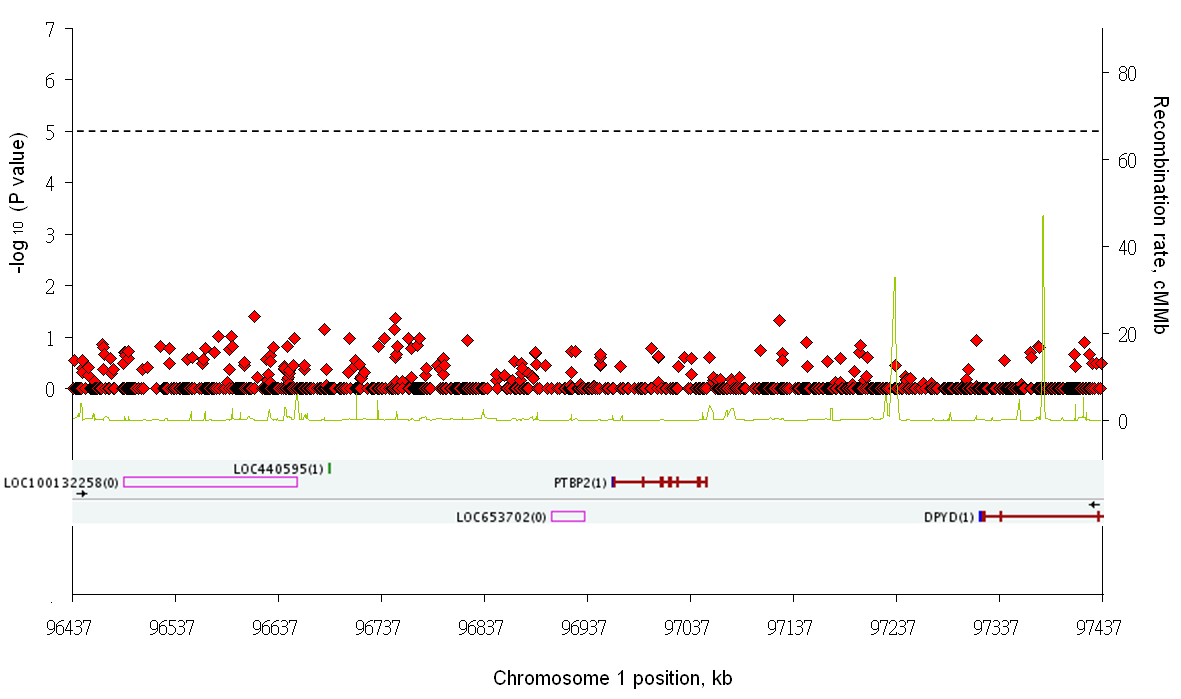


(B) *PLXNA2*

(C) *ZNF804A*


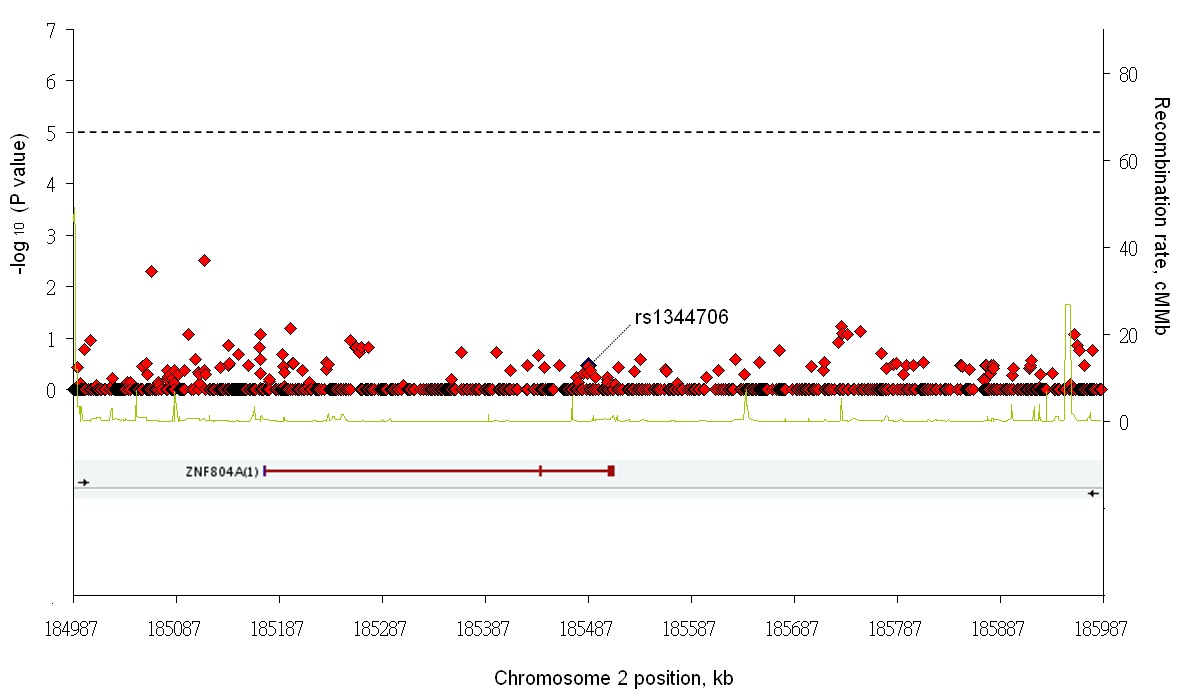


(D) *FXR1*


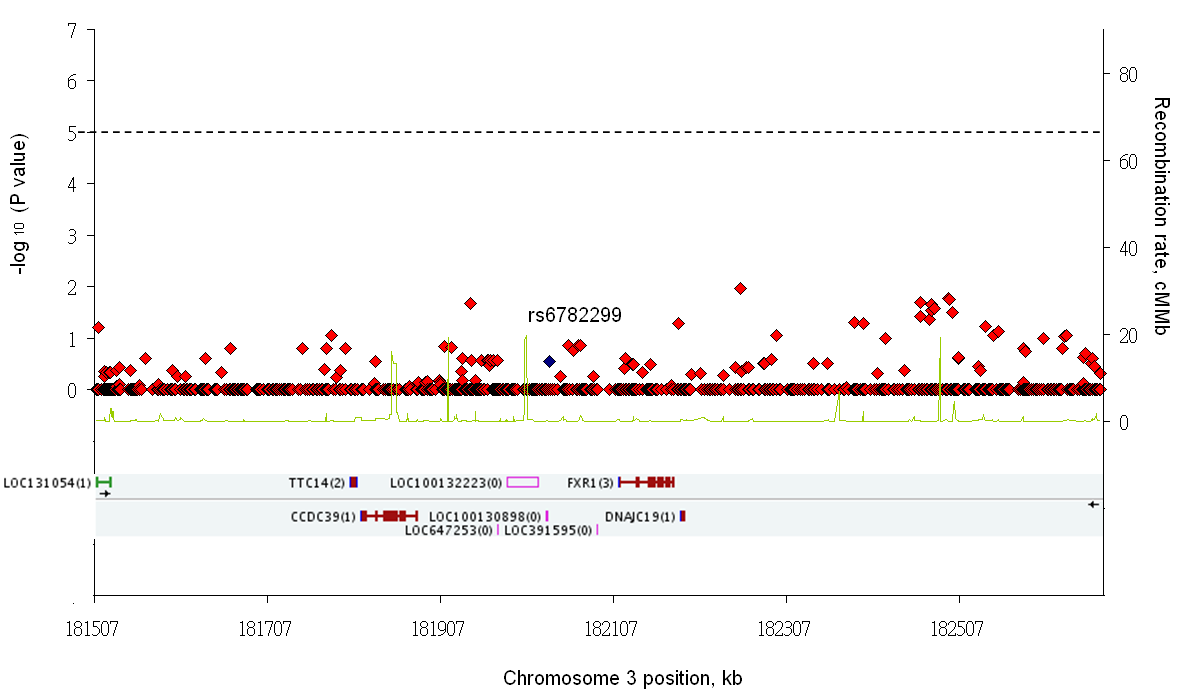


(E) MHC region/ *SLC17A1/ SLC17A3/ BTN2A2/ HIST1H2BJ/ PRSS16/ POM121L2/ ZNF184/ PGBD1*


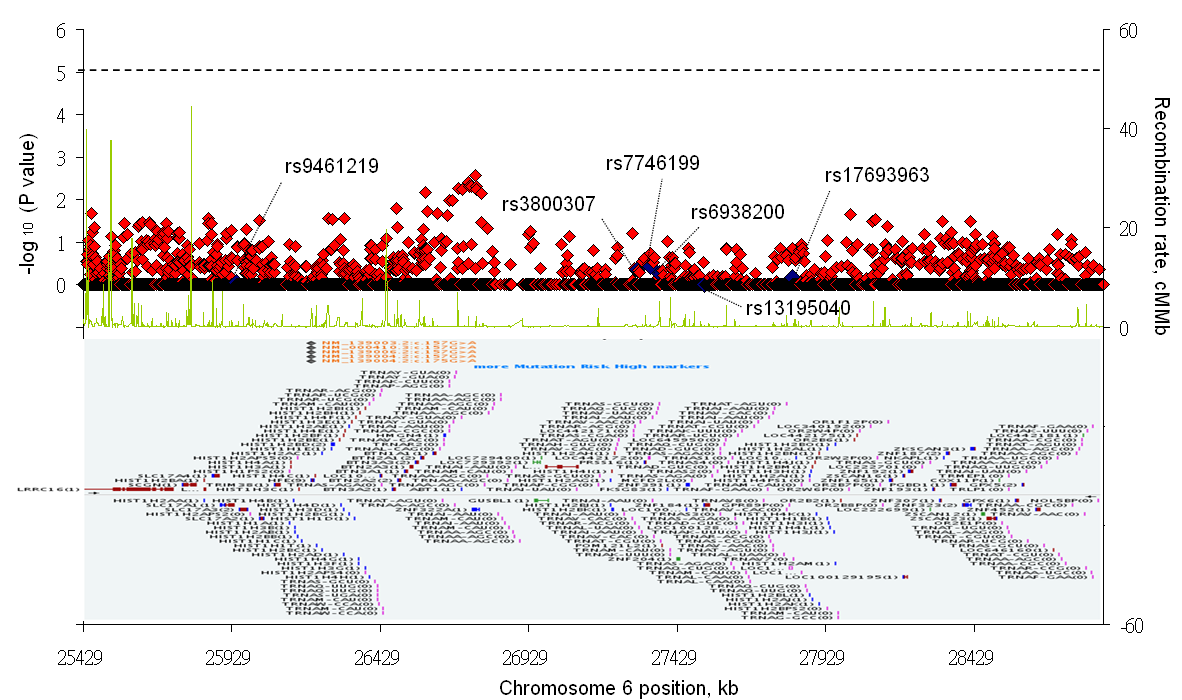


(F) MHC region/ *NOTCH4/ HLA-DQA1*


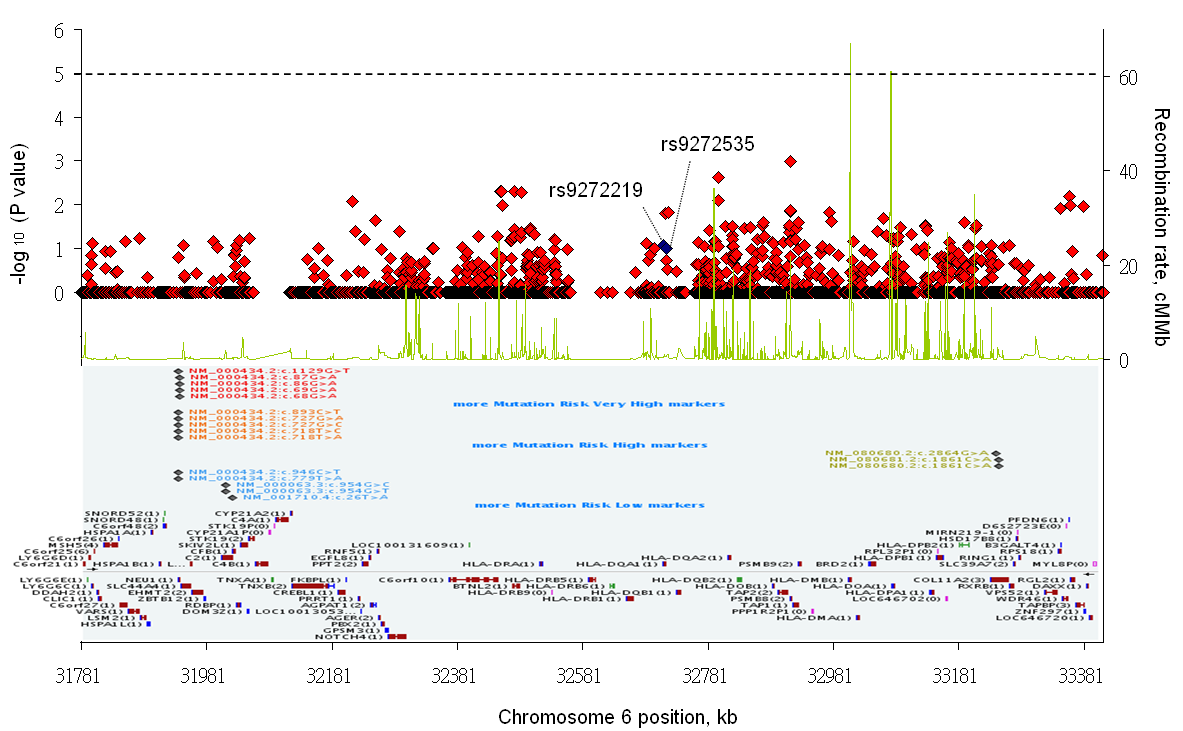


(G) *RELN*


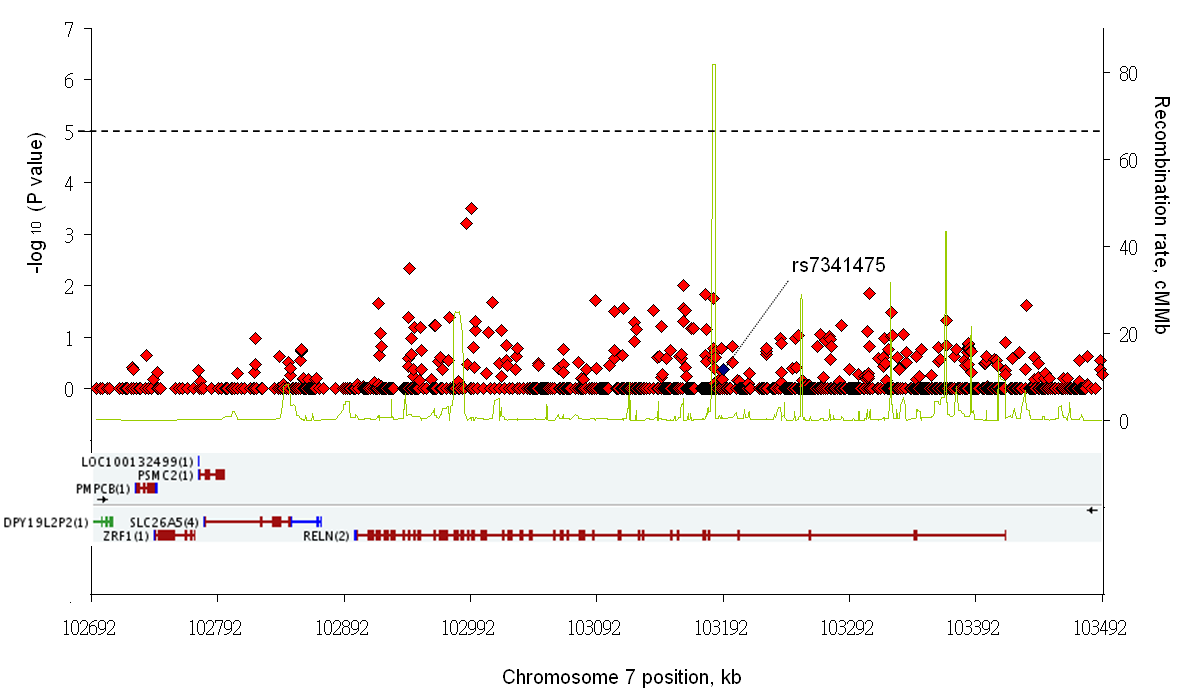


(H) *SMARCA2*


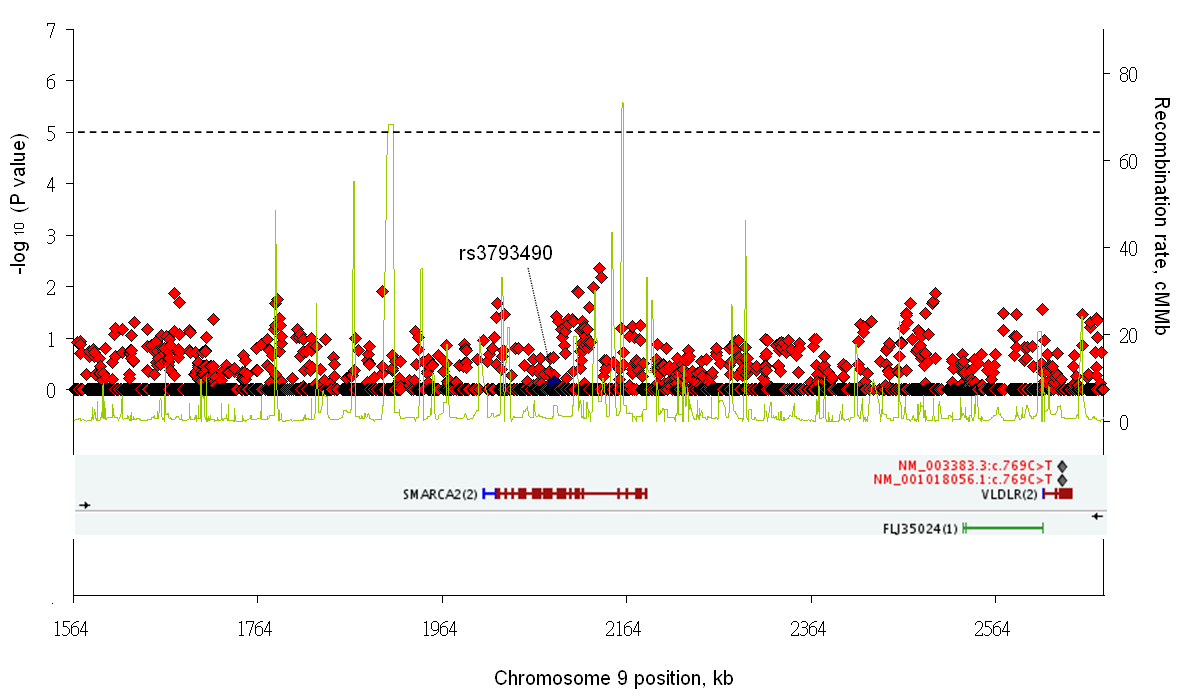


(I) *PLAA*


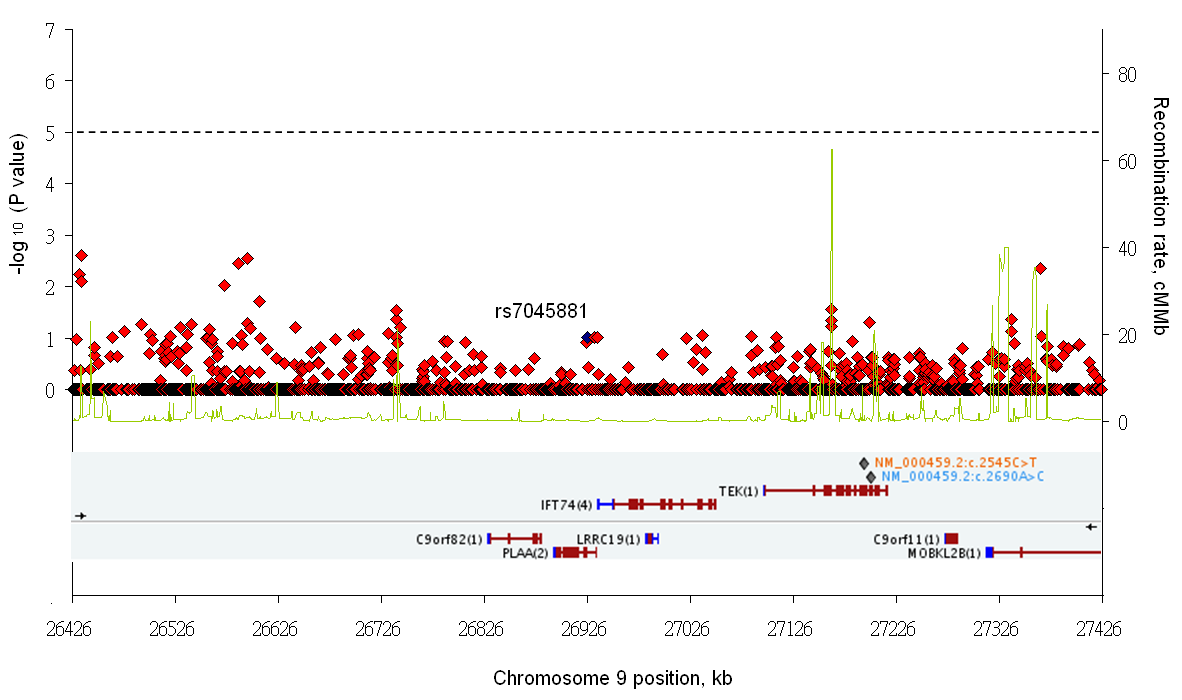


(J) *ANK3*


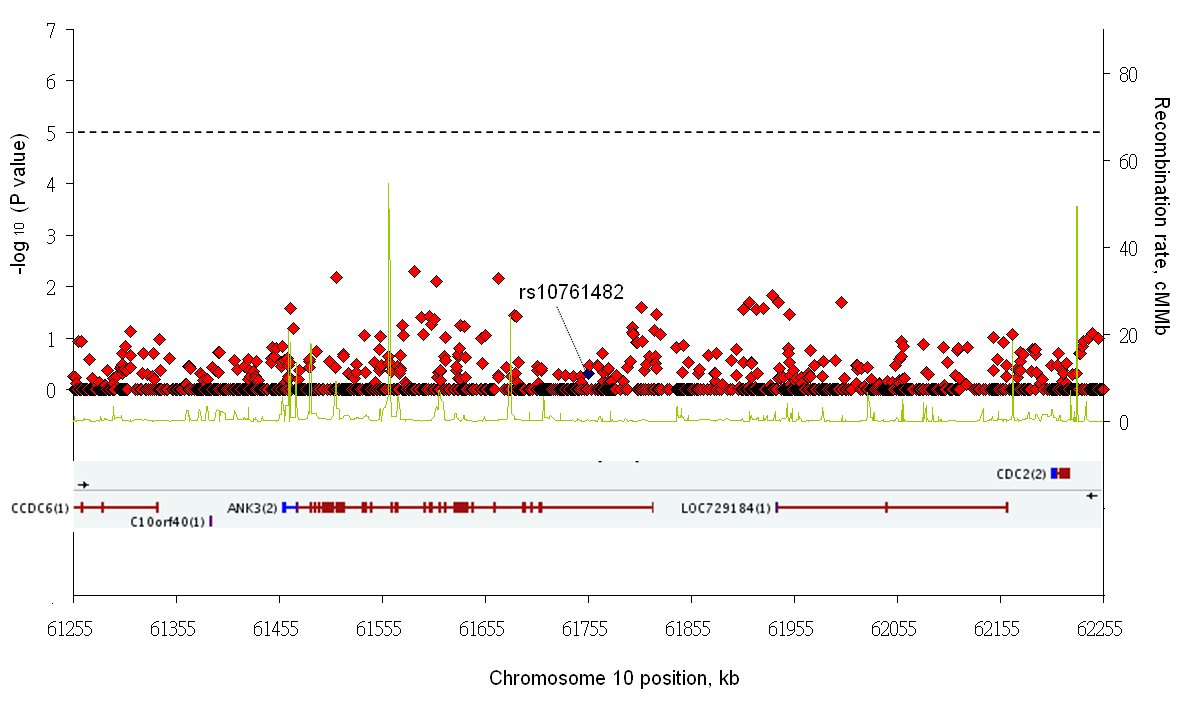


(K) Intergenic region on 11p14.1


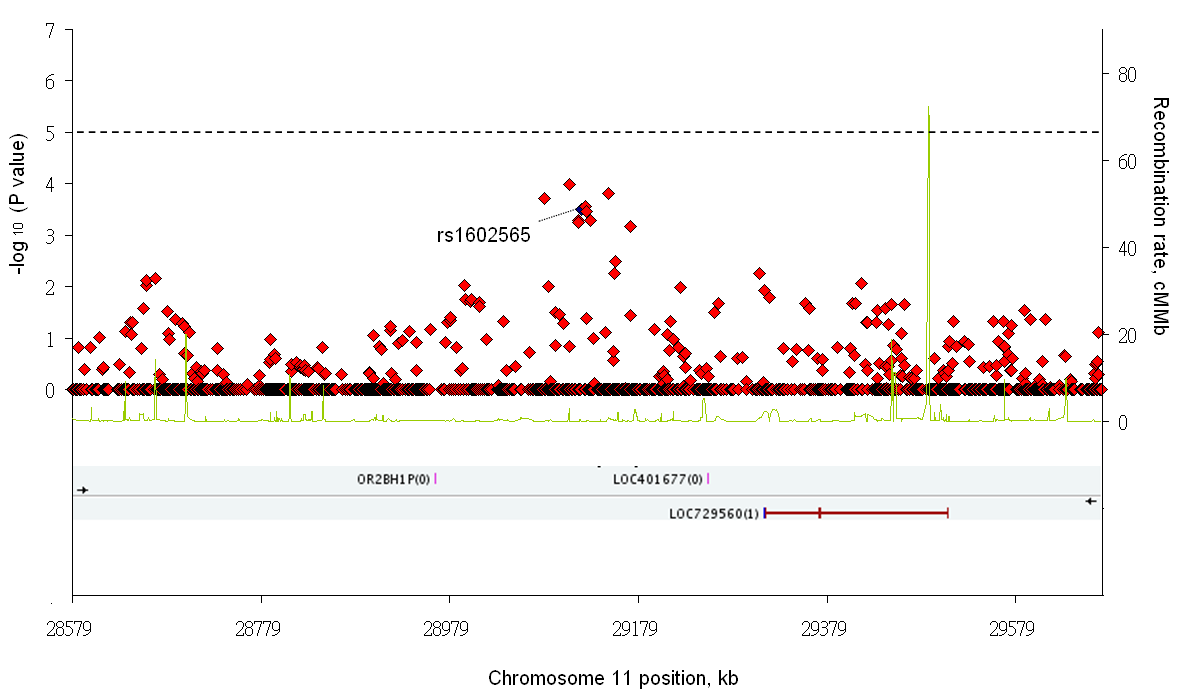


(L) *NRGN*/ I1 of *HEPACAM*


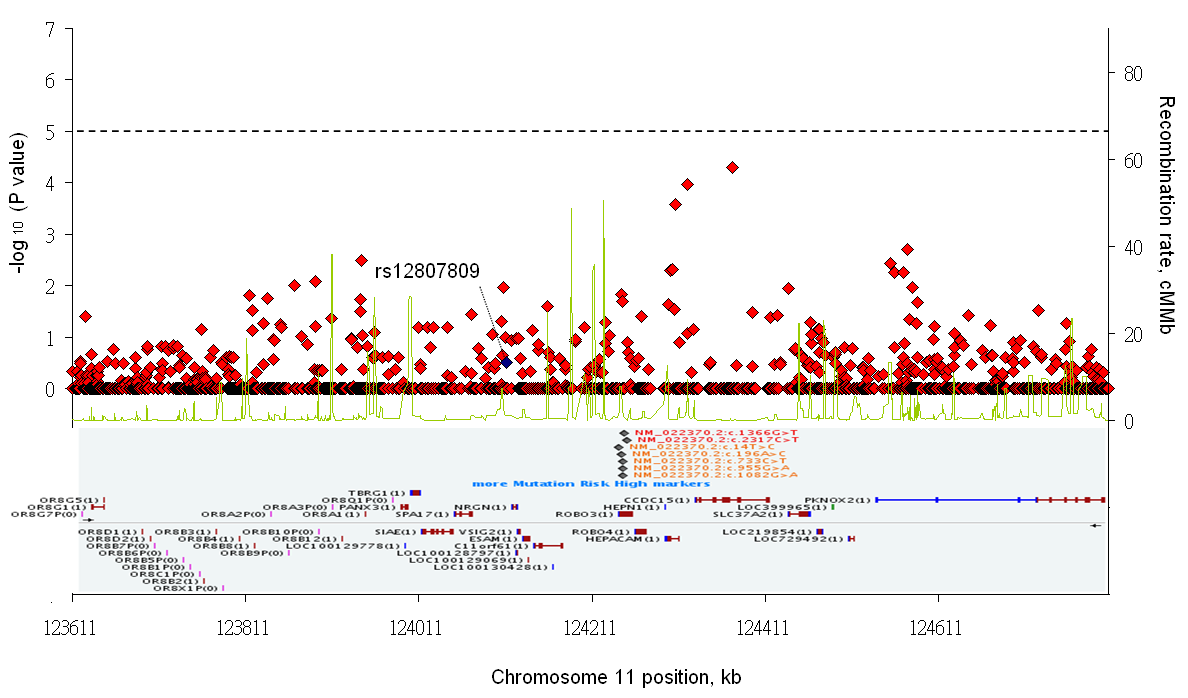


(M) Intergenic region on 16p13.2


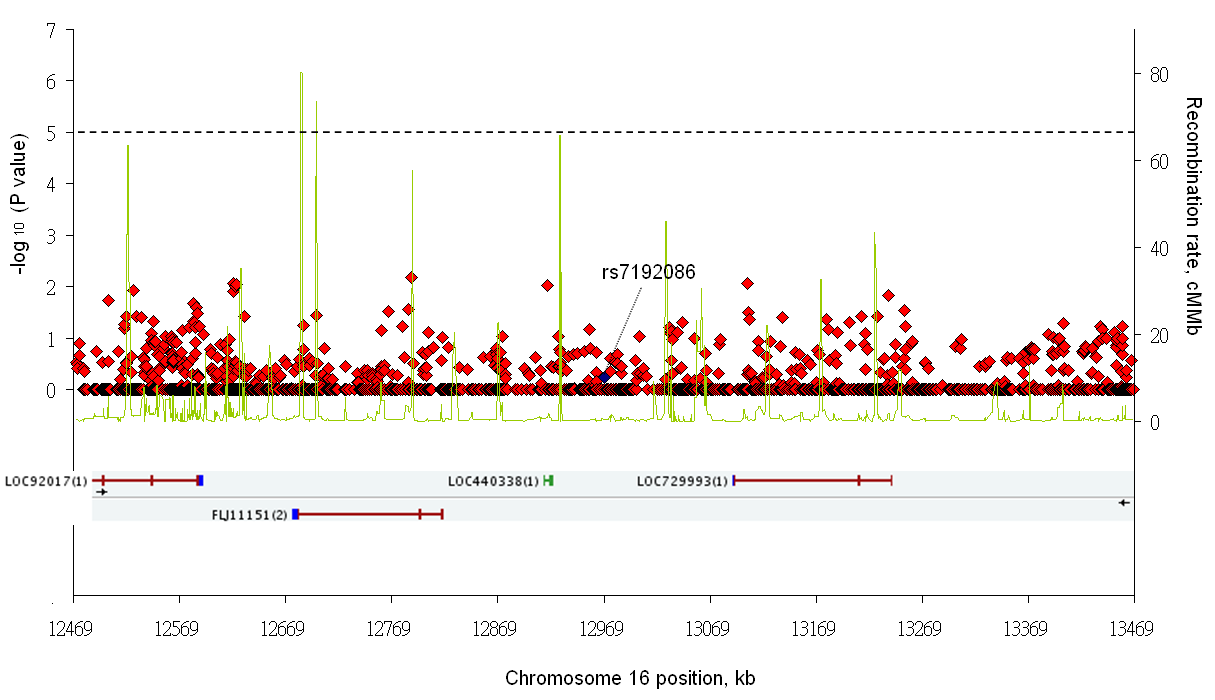


(N) *ACSM1*


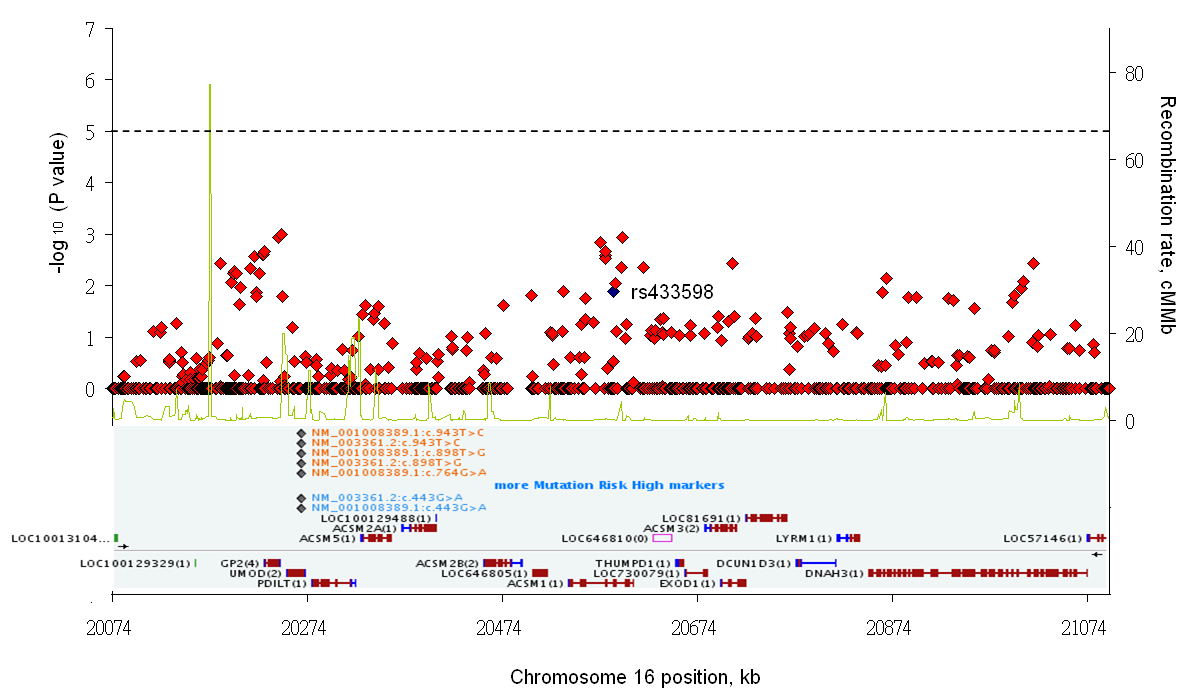


(O) *TCF4*


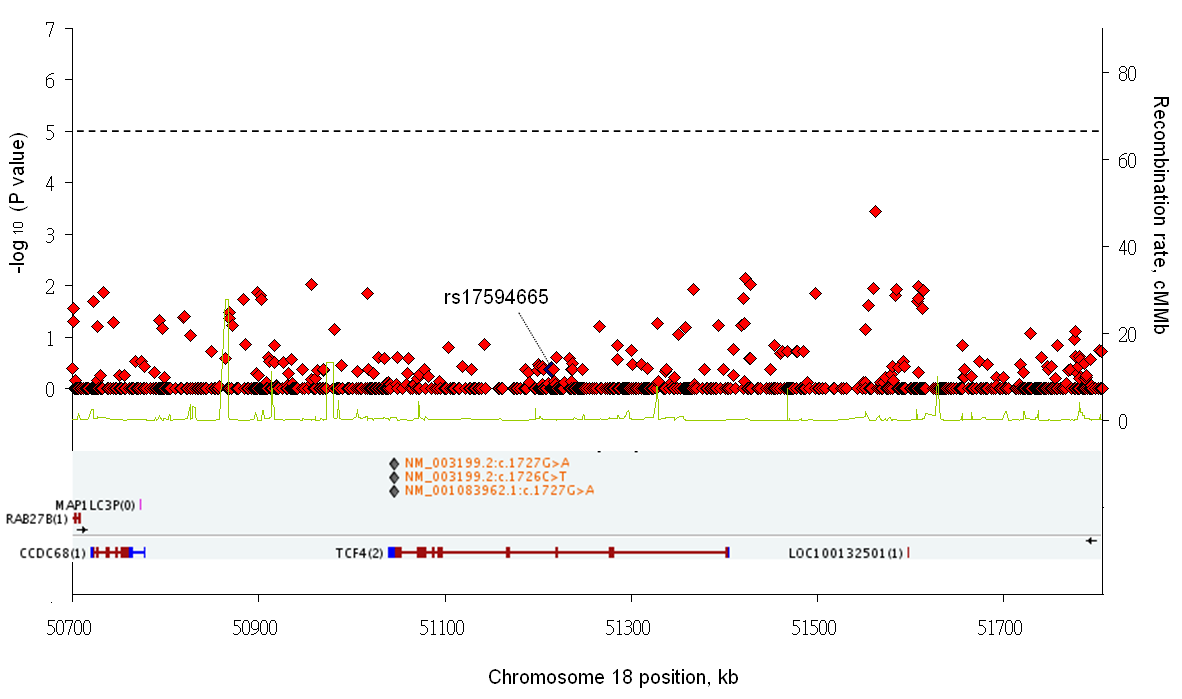

Supplement: Figure S5 — Comparisons to previous GWAS. For each of the (A) PTBP2, (B) PLXNA2, (C) ZNF804A, (D) FXR1, (E) MHC region/SLC17A1/SLC17A3/BTN2A2/HIST1H2BJ/PRSS16/POM121L2/ZNF184/PGBD1, (F) MHC region/NOTCH4/HLA-DQA1, (G) RELN, (H) SMARCA2, (I) PLAA, (J) ANK3, (K) Intergenic region on 11p14.1, (L) NRGN/I1 of HEPACAM, (M) Intergenic region on 16p13.2, (N) ACSM1, (O) TCF4, the −log10 P-values from primary scan are ploted as a function of genomic position (NCBI Build 36). The reported SNPs in previous GWAS are denoted by blue diamonds. Estimated recombination rates (right y axis) based on the Chinese HapMap population is plotted to reflect the local linkage disequilibrium structure around the significant SNPs. Gene annotations and number of transcripts were taken from NCBI. (DOC) [file pone.0033598.s005.doc]
