# Supplementary material for: Genome-Wide Association Study of Treatment Refractory Schizophrenia in Han Chinese
Source: PLoS One. 2012 Mar 27;7(3):e33598. doi: 10.1371/journal.pone.0033598 (PMC3313922; doi:10.1371/journal.pone.0033598)

**Supplementary Figure 6** Quantile-quantile (QQ) plots.

QQ plot is shown for the trend test. *P*-values are based on the 694,436SNPs which passed quality filters from 522 cases and 806 controls. The upper and lower boundaries of the 95% confidence bands are represented by the blue lines.


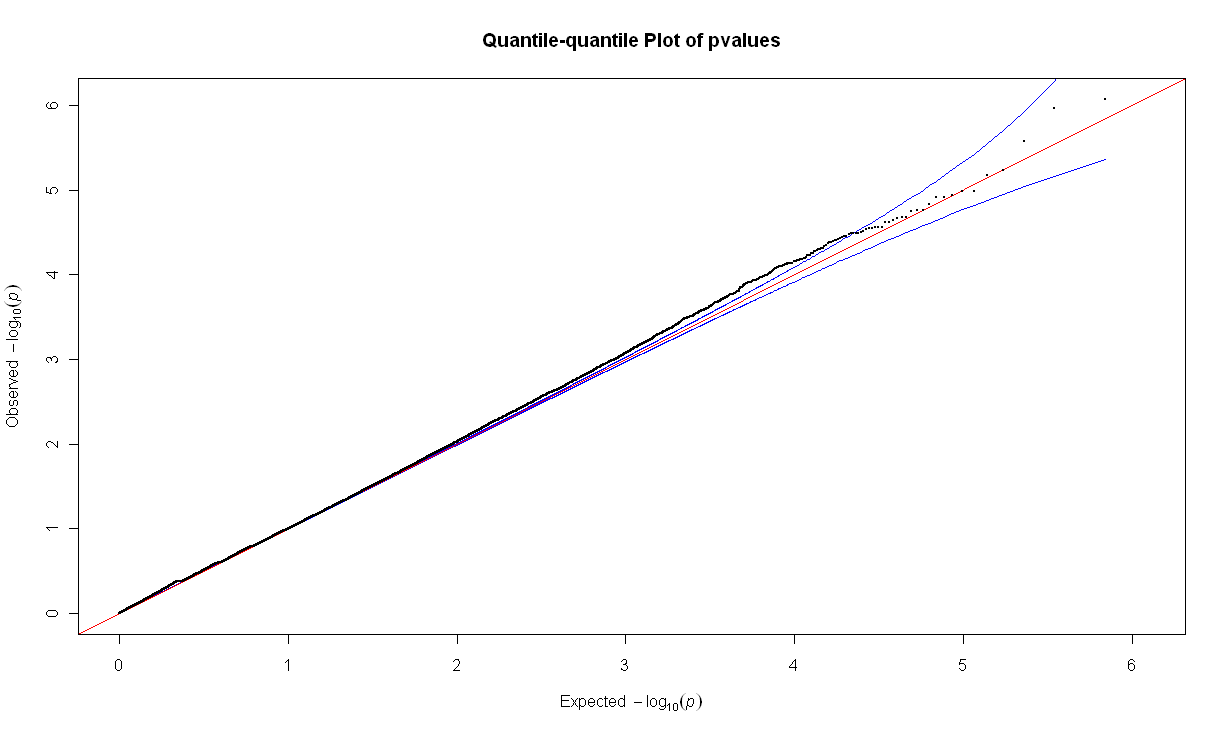

Supplement: Figure S6 — Quantile-quantile (QQ) plots. QQ plot is shown for the trend test. P-values are based on the 694,436SNPs which passed quality filters from 522 cases and 806 controls. The upper and lower boundaries of the 95% confidence bands are represented by the blue lines. (DOCX) [file pone.0033598.s006.docx]
