# Supplementary material for: Genome-Wide Association Study of Treatment Refractory Schizophrenia in Han Chinese
Source: PLoS One. 2012 Mar 27;7(3):e33598. doi: 10.1371/journal.pone.0033598 (PMC3313922; doi:10.1371/journal.pone.0033598)
Supplement: Table S1 — Quality control of the genotyping results. Breakdown of the number (N) of SNPs and samples which passed the QC filter. (DOCX) [file pone.0033598.s007.docx]

**Supplementary Table 1** Quality control of the genotyping results.

Breakdown of the number (N) of SNPs and samples which passed the QC filter

|  | Affymetrix SNP 6.0 | |
| --- | --- | --- |
|  | SNPs on chr1~22 | Individuals |
| N at start of QC | 868,114 | 1,328 |
| N dropped during exclusion steps: |  |  |
| 1.  SNPs with non-polymorphic in case and control | 72,654 | - |
| 2.   SNPs with overall call rate < 0.98 | 74,453 | - |
| 3.   SNPs with overall MAF <0.05 & overall call rate < 0.99 | 12,819 | - |
| 4.   SNPs with HWE for controls (*P* < 10^-4^) | 13,752 | - |
|  |  |  |
| N at end of QC | 694,436 | 1,328 |
