# Supplementary material for: Genome-Wide Association Study of Treatment Refractory Schizophrenia in Han Chinese
Source: PLoS One. 2012 Mar 27;7(3):e33598. doi: 10.1371/journal.pone.0033598 (PMC3313922; doi:10.1371/journal.pone.0033598)
Supplement: Table S2 — Concordance rates for the 19 SNPs with significant associations in the initial GWA analysis. (DOCX) [file pone.0033598.s008.docx]

**Supplementary Table 2** Concordance rates for the 19 SNPs with significant associations in the initial GWA analysis.

| SNP | Chromosomes | Position | Affymetrix SNP 6.0 | | Sequenom MassARRAY | | Concordance rate* |
| --- | --- | --- | --- | --- | --- | --- | --- |
|  |  |  | Call rate | | Call rate | |  |
|  |  |  | Case | Control | Case | Control |  |
| rs10218843 | 1 | 158892685 | 1.000 | 1.000 | 0.999 | 1.000 | 100.00% |
| rs11265461 | 1 | 158896767 | 0.990 | 0.985 | 0.996 | 1.000 | 99.85% |
| rs4952667 | 2 | 43226138 | 0.993 | 0.977 | 0.999 | 1.000 | 93.14% |
| rs230529 | 4 | 103676448 | 1.000 | 1.000 | 0.991 | 0.992 | 100.00% |
| rs230505 | 4 | 103700386 | 0.999 | 1.000 |  | no call | 100.00% |
| rs4699030 | 4 | 103722862 | 0.998 | 1.000 | 0.992 | 0.998 | 99.54% |
| rs461409 | 5 | 97957866 | 0.978 | 0.992 | 0.930 | 0.981 | 98.70% |
| rs9358039 | 6 | 14680938 | 0.995 | 0.992 | 0.999 | 1.000 | 92.81% |
| rs6973516 | 7 | 63835240 | 0.988 | 1.000 | 0.083 | 0.094 | 30.70% |
| rs12533497 | 7 | 91495608 | 1.000 | 1.000 | 0.999 | 1.000 | 99.92% |
| rs739617 | 7 | 111298102 | 0.993 | 0.998 | 0.997 | 1.000 | 99.92% |
| rs17158926 | 7 | 111298199 | 0.999 | 1.000 | 0.999 | 1.000 | 99.92% |
| rs17158930 | 7 | 111298374 | 1.000 | 1.000 | 0.997 | 1.000 | 100.00% |
| rs9314462 | 8 | 2501291 | 0.999 | 1.000 | 0.994 | 0.996 | 100.00% |
| rs9646303 | 16 | 86019470 | 0.976 | 0.994 | 0.996 | 0.998 | 99.92% |
| rs11673496 | 19 | 22581270 | 1.000 | 1.000 | 0.999 | 1.000 | 99.85% |
| rs11673589 | 19 | 22581559 | 1.000 | 1.000 | 0.804 | 0.855 | 73.27% |
| rs13049286 | 21 | 42049868 | 0.999 | 1.000 | 0.999 | 1.000 | 100.00% |
| rs3827219 | 21 | 42053555 | 1.000 | 1.000 | 0.999 | 1.000 | 100.00% |

* Concordance rates were calculated based on the concordance of genotyping results between the Affymetrix SNP 6.0 and the Sequenom Mass

Array; SNPs with concordance rate less than 0.98 were not analyzed further.
