# Supplementary material for: Genome-Wide Association Study of Treatment Refractory Schizophrenia in Han Chinese
Source: PLoS One. 2012 Mar 27;7(3):e33598. doi: 10.1371/journal.pone.0033598 (PMC3313922; doi:10.1371/journal.pone.0033598)
Supplement: Table S3 — Multipoint/haplotype analysis of the clusters on chromosome 1 (A), chromosome 4 (B), and chromosome 7(C). (DOCX) [file pone.0033598.s009.docx]

**Supplementary Table 3** Multipoint/haplotype analysis of the clusters on chromosome 1 (A), chromosome 4 (B), and chromosome 7(C).

1. chromosome 1

|  | rs2295614 | rs11265455 | rs10218843 | rs4656923 | rs11265461 | Hap-Score | p-val | Hap-Freq | Control freq | Case Freq | Global Score *p*-value | Global Simulation *p*-value |
| --- | --- | --- | --- | --- | --- | --- | --- | --- | --- | --- | --- | --- |
| 1 | A | G | T | C | T | -3.165110211 | 0.001550242 | 0.312026311 | 0.334788438 | 0.277346298 |  |  |
| 2 | A | A | T | A | T | -2.466996707 | 0.013625161 | 0.221059963 | 0.236890208 | 0.196932228 |  |  |
| 3 | A | A | T | C | T | 1.851275179 | 0.064129975 | 0.013298522 | 0.009730147 | 0.018419215 |  |  |
| 4 | A | A | C | C | C | 2.356226785 | 0.018461643 | 0.053340298 | 0.045003228 | 0.066060491 |  |  |
| 5 | T | A | C | C | C | 3.467189856 | 0.00052593 | 0.382312531 | 0.356260325 | 0.422375092 |  |  |
|  |  |  |  |  |  |  |  |  |  |  | 3.00E-05 | 2.00E-05 |

1. Chromosome 4

|  | rs230532 | rs230531 | rs230529 | rs230525 | rs4648006 | rs230520 | rs230519 | rs93059 | rs230504 | rs230493 | rs1598861 | rs230500 | rs4699030 |
| --- | --- | --- | --- | --- | --- | --- | --- | --- | --- | --- | --- | --- | --- |
| 1 | T | A | C | A | C | T | G | A | C | A | G | C | G |
| 2 | T | A | C | A | C | T | G | A | C | A | T | C | G |
| 3 | T | A | C | A | C | T | G | G | C | A | T | C | G |
| 4 | T | A | T | A | T | T | G | G | C | A | T | C | C |
| 5 | A | G | T | G | C | C | A | G | T | T | T | T | C |
|  |  |  |  |  |  |  |  |  |  |  |  |  |  |

|  | rs1610152 | rs1598859 | rs3774959 | rs4648055 | rs4648068 | rs4648110 | Hap-Score | p-val | Hap-Freq | Control Freq | Case Freq | Global Score p-value | Global Simulation p-value |
| --- | --- | --- | --- | --- | --- | --- | --- | --- | --- | --- | --- | --- | --- |
| 1 | G | A | G | C | A | T | -3.43247 | 0.000598 | 0.109801 | 0.12652 | 0.084018 |  |  |
| 2 | G | A | G | C | A | T | -2.25529 | 0.024115 | 0.319396 | 0.335633 | 0.295026 |  |  |
| 3 | G | A | G | C | A | A | -1.65405 | 0.098117 | 0.056724 | 0.062655 | 0.047619 |  |  |
| 4 | C | A | G | C | A | T | 2.000273 | 0.045471 | 0.064031 | 0.056452 | 0.075238 |  |  |
| 5 | G | G | A | T | G | T | 3.729536 | 0.000192 | 0.438762 | 0.410046 | 0.482841 |  |  |
|  |  |  |  |  |  |  |  |  |  |  |  | 2.00E-05 | 3.00E-05 |

(C) Chromosome 7

|  | rs2074130 | rs12154389 | rs2074133 | rs2074134 | rs7788189 | rs739617 | rs17158926 | rs17158930 | rs10253643 |
| --- | --- | --- | --- | --- | --- | --- | --- | --- | --- |
| 1 | C | G | G | C | C | G | T | A | G |
| 2 | C | A | A | C | C | G | T | A | A |
| 3 | C | G | A | C | C | G | T | A | A |
| 4 | T | G | G | C | C | G | T | A | G |
| 5 | C | A | A | T | G | A | A | G | A |
|  |  |  |  |  |  |  |  |  |  |
|  |  |  |  |  |  |  |  |  |  |
|  |  |  |  |  |  |  |  |  |  |
|  | Hap-Score | p-val | Hap-Freq | Control Freq | Case Freq | Global Score p-value | Global Simulation p-value |  |  |
| 1 | -1.80003764 | 0.0718547 | 0.481944386 | 0.4963814 | 0.4609169 |  |  |  |  |
| 2 | -1.54580794 | 0.12215096 | 0.203633145 | 0.21348528 | 0.1885228 |  |  |  |  |
| 3 | -0.54000949 | 0.58919049 | 0.034721166 | 0.03623313 | 0.0323989 |  |  |  |  |
| 4 | 0.80084743 | 0.42321998 | 0.100619788 | 0.09658983 | 0.1056754 |  |  |  |  |
| 5 | 4.22220969 | 2.42E-05 | 0.153543778 | 0.13009615 | 0.1895164 |  |  |  |  |
|  |  |  |  |  |  | 1.32E-03 | 1.12E-03 |  |  |
