# Supplementary material for: Genome-Wide Association Study of Treatment Refractory Schizophrenia in Han Chinese
Source: PLoS One. 2012 Mar 27;7(3):e33598. doi: 10.1371/journal.pone.0033598 (PMC3313922; doi:10.1371/journal.pone.0033598)
Supplement: Table S4 — Testing TRS association results with schizophrenia. (DOCX) [file pone.0033598.s010.docx]

**Supplementary Table 4** Testing TRS association results with schizophrenia.

|  |  | TRS case n=522, controls n = 806 | | | | | | | |  | SZ case n=1982, controls n = 2000 | | | | | | | |
| --- | --- | --- | --- | --- | --- | --- | --- | --- | --- | --- | --- | --- | --- | --- | --- | --- | --- | --- |
| chr | SNP | Call Rate | |  | RAF | |  | log_trend | max_p |  | Call Rate | |  | RAF | |  | log_trend | max_*p* |
|  |  | CTL | TRS |  | CTL | TRS |  |  |  |  | CTL | TRS |  | CTL | TRS |  |  |  |
| 1 | rs10218843 | 1.000 | 1.000 |  | 0.407 | 0.495 |  | 5.172 | 5.172 |  | 0.991 | 0.997 |  | 0.457 | 0.459 |  | 0.072 | 0.128 |
| 1 | rs11265461 | 0.990 | 0.985 |  | 0.411 | 0.500 |  | 5.229 | 5.229 |  | 0.991 | 0.997 |  | 0.459 | 0.462 |  | 0.091 | 0.211 |
| 3 | rs977324 | 0.993 | 0.998 |  | 0.684 | 0.612 |  | 3.978 | 5.654 |  | 0.996 | 0.995 |  | 0.654 | 0.658 |  | 0.138 | 0.727 |
| 4 | rs230529 | 1.000 | 1.000 |  | 0.528 | 0.430 |  | 5.970 | 6.052 |  | 0.990 | 0.994 |  | 0.481 | 0.460 |  | 1.202 | 1.202 |
| 4 | rs4699030 | 0.998 | 1.000 |  | 0.470 | 0.568 |  | 6.075 | 6.119 |  | 0.997 | 0.993 |  | 0.485 | 0.461 |  | 1.501 | 1.501 |
| 4 | rs11932853 | 0.993 | 1.000 |  | 0.438 | 0.425 |  | 0.304 | 5.017 |  | 0.997 | 0.994 |  | 0.428 | 0.417 |  | 0.490 | 0.615 |
| 5 | rs461409 | 0.978 | 0.992 |  | 0.207 | 0.136 |  | 5.580 | 5.580 |  | 0.997 | 0.993 |  | 0.181 | 0.182 |  | 0.043 | 0.522 |
| 7 | rs12533497 | 1.000 | 1.000 |  | 0.929 | 0.878 |  | 4.983 | 5.020 |  | 0.992 | 0.996 |  | 0.917 | 0.908 |  | 0.786 | 0.806 |
| 7 | rs739617 | 0.993 | 0.998 |  | 0.130 | 0.191 |  | 4.836 | 5.296 |  | 0.992 | 0.995 |  | 0.151 | 0.146 |  | 0.273 | 0.273 |
| 7 | rs17158926 | 0.999 | 1.000 |  | 0.135 | 0.193 |  | 4.399 | 5.048 |  | 0.997 | 0.994 |  | 0.152 | 0.148 |  | 0.163 | 0.163 |
| 7 | rs17158930 | 1.000 | 1.000 |  | 0.866 | 0.807 |  | 4.512 | 5.181 |  | 0.992 | 0.997 |  | 0.847 | 0.851 |  | 0.194 | 0.209 |
| 8 | rs9314462 | 0.999 | 1.000 |  | 0.199 | 0.266 |  | 4.276 | 5.062 |  | 0.992 | 0.985 |  | 0.227 | 0.255 |  | 2.520 | 2.520 |
| 11 | rs10791335 | 0.999 | 1.000 |  | 0.350 | 0.420 |  | 3.710 | 5.791 |  | 0.989 | 0.986 |  | 0.366 | 0.379 |  | 0.583 | 0.626 |
| 16 | rs9646303 | 0.976 | 0.994 |  | 0.409 | 0.496 |  | 4.940 | 5.204 |  | 0.995 | 0.993 |  | 0.467 | 0.477 |  | 0.411 | 0.777 |
| 19 | rs11673496 | 1.000 | 1.000 |  | 0.268 | 0.193 |  | 4.752 | 5.487 |  | 0.991 | 0.994 |  | 0.220 | 0.225 |  | 0.211 | 0.211 |
| 21 | rs13049286 | 0.999 | 1.000 |  | 0.986 | 0.959 |  | 4.912 | 5.077 |  | 0.998 | 0.994 |  | 0.971 | 0.965 |  | 0.822 | 1.455 |
| 21 | rs3827219 | 1.000 | 1.000 |  | 0.014 | 0.042 |  | 4.910 | 5.064 |  | 0.997 | 0.995 |  | 0.031 | 0.035 |  | 0.648 | 1.253 |

| TRS: treatment refractory schizophrenia |
| --- |
| SZ: schizophrenia |
| RA: Risk allele, the allele with higher frequency in schizophrenia as compared with controls; |
