# Supplementary material for: Genome-Wide Association Study of Treatment Refractory Schizophrenia in Han Chinese
Source: PLoS One. 2012 Mar 27;7(3):e33598. doi: 10.1371/journal.pone.0033598 (PMC3313922; doi:10.1371/journal.pone.0033598)
Supplement: Table S5 — SNPs showing suggestive significant associations adjusted using genomic control. (DOCX) [file pone.0033598.s011.docx]

**Supplementary Table 5** SNPs showing suggestive significant associations adjusted using genomic control.

| **Chr** | **SNP_ID** | **Position** | ***P* best** | ***P* _trend_** | ***P* _genomic control_ (trend)** | ***P* _permutation_ (trend)** |
| --- | --- | --- | --- | --- | --- | --- |
| 1 | rs10218843 | 158892685 | 6.73E-06 (trend) | 6.73E-06 | 2.86E-05 | 6.94E-06 |
| 1 | rs11265461 | 158896767 | 5.9E-06 (trend) | 5.90E-06 | 2.55E-05 | 5.91E-06 |
| 3 | rs977324 | 65267001 | 2.22E-06 (rec) | 1.05E-04 | 3.12E-04 | 1.02E-04 |
| 4 | rs230529 | 103676448 | 8.87E-07 (allele) | 1.07E-06 | 5.79E-06 | 1.13E-06 |
| 4 | rs4699030 | 103722862 | 7.61E-07 (allele) | 8.41E-07 | 4.69E-06 | 8.10E-07 |
| 4 | rs11932853 | 112243837 | 9.61E-06 (geno) | 0.49627 | 0.52712 | 0.49540 |
| 5 | rs461409 | 97957866 | 2.63E-06 (trend) | 2.63E-06 | 1.26E-05 | 2.06E-06 |
| 7 | rs12533497 | 91495608 | 9.56E-06 (allele) | 1.04E-05 | 4.17E-05 | 9.90E-06 |
| 7 | rs739617 | 111298102 | 5.06E-06 (rec) | 1.46E-05 | 5.59E-05 | 1.35E-05 |
| 7 | rs17158926 | 111298199 | 8.94E-06 (rec) | 3.99E-05 | 1.34E-04 | 3.76E-05 |
| 7 | rs17158930 | 111298374 | 6.6E-06 (dom) | 3.08E-05 | 1.07E-04 | 3.33E-05 |
| 8 | rs9314462 | 2501291 | 8.6768E-06 (rec) | 5.30E-05 | 1.72E-04 | 5.56E-05 |
| 11 | rs10791335 | 133244781 | 1.62E-06 (rec) | 1.95E-04 | 5.35E-04 | 1.82E-04 |
| 16 | rs9646303 | 86019470 | 6.25E-06 (dom) | 1.15E-05 | 4.54E-05 | 1.11E-05 |
| 19 | rs11673496 | 22581270 | 3.26E-06 (rec) | 1.77E-05 | 6.62E-05 | 2.05E-05 |
| 21 | rs13049286 | 42049868 | 8.37E-06 (allele) | 1.23E-05 | 4.81E-05 | 1.49E-05 |
| 21 | rs3827219 | 42053555 | 8.63E-06 (allele) | 1.23E-05 | 4.82E-05 | 1.38E-05 |
