# Supplementary material for: Genome-Wide Association Study of Treatment Refractory Schizophrenia in Han Chinese
Source: PLoS One. 2012 Mar 27;7(3):e33598. doi: 10.1371/journal.pone.0033598 (PMC3313922; doi:10.1371/journal.pone.0033598)
Supplement: Table S6 — Previously reported loci and SNPs associated with schizophrenia. (DOCX) [file pone.0033598.s012.docx]

**Supplementary Table 6** Previously reported loci and SNPs associated with schizophrenia

|  |  |  | Position | Previous GWAS | | |  | This study* | | |
| --- | --- | --- | --- | --- | --- | --- | --- | --- | --- | --- |
| Genes/loci | Chr | SNP | (NCBI build 36.3) | Allele | *P* value | ref |  | Allele | *P* value (best) | *P* value (trend) |
| PTBP2 | 1 | rs7544736 | 96936931 | G/A | 5.7 x 10^-7^ | 24 |  | - | - | - |
| PLXNA2 | 1 | rs2262269 | 206134274 | - | - | - |  | A/C | 2.0 x 10^-4^ | 2.0 x 10^-4^ |
|  |  | rs841865 | 206292532 | G/A | 0.008 | 25 |  | - | - | - |
|  |  | rs752016 | 206304300 | T/C | 0.006 | 25 |  | A/G | 0.034 | 0.034 |
|  |  | rs1327175 | 206313757 | C/G | 0.02 | 25 |  | - | - | - |
|  |  | rs2498028 | 206321936 | T/C | 0.001 | 25 |  | - | - | - |
|  |  | rs4406633 | 206417575 | - | - | - |  | A/G | 6.9 x 10^-4^ | 8.5 x 10^-4^ |
|  |  | rs11590686 | 206434824 | - | - | - |  | A/G | 2.3 x 10^-5^ | 7.0 x 10^-5^ |
| ZNF804A | 2 | rs1344706 | 185486673 | T/G | 1.6 x 10^-7^ | 18 |  | G/T | 0.342 | 0.584 |
| FXR1 | 3 | rs7640601 | 182007458 | G/C | 7.3 x 10^-7^ | 24 |  | - | - | - |
|  |  | rs9869882 | 182014635 | A/G | 3.8 x 10^-7^ | 24 |  | - | - | - |
|  |  | rs9838229 | 182015945 | C/A | 2.1 x 10^-7^ | 24 |  | - | - | - |
|  |  | rs4488266 | 182018099 | C/T | 3.8 x 10^-7^ | 24 |  | - | - | - |
|  |  | rs2139551 | 182019059 | A/G | 1.8 x 10^-7^ | 24 |  | - | - | - |
|  |  | rs10937048 | 182022086 | A/G | 3.9 x 10^-7^ | 24 |  | - | - | - |
|  |  | rs6782299 | 182033396 | G/T | 1.4 x 10^-7^ | 24 |  | G/T | 0.280 | 0.751 |
|  |  | rs1879248 | 182033908 | C/T | 1.5 x 10^-7^ | 24 |  | - | - | - |
|  |  | rs1915104 | 182039598 | C/A | 1.5 x 10^-7^ | 24 |  | - | - | - |
|  |  | rs6767560 | 182048915 | G/T | 4.0 x 10^-7^ | 24 |  | - | - | - |
|  |  | rs2337743 | 182059367 | T/A | 1.6 x 10^-7^ | 24 |  | - | - | - |
|  |  | rs1607678 | 182067412 | C/T | 1.7 x 10^-7^ | 24 |  | - | - | - |
|  |  | rs1010471 | 182173786 | A/G | 6.5 x 10^-7^ | 24 |  | - | - | - |
| MHC region/SLC17A1 | 6 | rs6939997 | 25929203 | T/C | 1.9  x 10^-7^ | 20 |  | - | - | - |
|  |  | rs13199775 | 25936761 | T/A | 1.2 x 10^-7^ | 20 |  | - | - | - |
|  |  | rs9461219 | 25944906 | G/C | 4.7 x 10^-7^ | 20 |  | C/G | 0.886 | 0.886 |
|  |  |  |  |  |  |  |  |  |  |  |
| MHC region/ SLC17A3 | 6 | rs9467626 | 25981725 | A/C | 1.7 x 10^-7^ | 20 |  | - | - | - |
| MHC region/BTN3A2 | 6 | rs2072806 | 26493072 | G/C | 9.3 x 10^-7^ | 20 |  | - | - | - |
| MHC region/BTN2A2 | 6 | rs2072803 | 26500494 | C/G | 8.2 x 10^-7^ | 20 |  | - | - | - |
| MHC region | 6 | rs6904071 | 27155235 | A/G | 1.8 x 10^-8^ | 20,24 |  | - | - | - |
|  |  | rs926300 | 27167422 | T/A | 1.1 x 10^-8^ | 20,24 |  | - | - | - |
| MHC region/HIST1H2BJ | 6 | rs6913660 | 27199404 | A/C | 1.1 x 10^-9^, 2.4 x 10^-8^ | 20,21,24 |  | - | - | - |
| MHC region | 6 | rs13219181 | 27244204 | G/A | 1.3 x 10^-8^ | 20,24 |  | - | - | - |
|  |  | rs13194053 | 27251862 | C/T | 9.5 x 10^-9^ | 20,24 |  | - | - | - |
| MHC region/ PRSS16 | 6 | rs13219354 | 27293643 | C/T | 1.3 x 10^-10^, 1.1 x 10^-7^ | 20 |  | - | - | - |
|  |  | rs3800307 | 27293771 | A/T | 4.4 x 10^-8^ | 20,24 |  | A/T | 0.440 | 0.440 |
|  |  | rs13212921 | 27313401 | T/C | 1.3 x 10^-7^ | 20 |  | - | - | - |
|  |  | rs6932590 | 27356910 | C/T | 1.4 x 10^-12^ , 7.1 x 10^-8^ | 20,21,24 |  | - | - | - |
|  |  | rs4452638 | 27337244 | A/G | 2.7 x 10^-7^ | 20 |  | - | - | - |
|  |  | rs6938200 | 27339129 | G/A | 3.0 x 10^-7^ | 20 |  | A/G | 0.378 | 0.378 |
| MHC region | 6 | rs3800316 | 27364081 | C/A | 3.8 x 10^-8^ | 20,24 |  | - | - | - |
|  |  | rs7746199 | 27369303 | T/C | 5.0 x 10^-8^ | 20,24 |  | A/G | 0.541 | 0.543 |
|  |  | rs3800318 | 27371620 | T/A | 6.4 x 10^-8^ | 20,24 |  | - | - | - |
| MHC region/ POM121L2 | 6 | rs16897515 | 27385999 | A/C | 1.8 x 10^-7^ | 20 |  | - | - | - |
| MHC region/ ZNF184 | 6 | rs13195040 | 27521903 | G/A | 2.5 x 10^-7^ | 20 |  | C/T | 0.980 | 0.980 |
| MHC region | 6 | rs10484399 | 27642507 | G/A | 3.5 x 10^-7^ | 20 |  | - | - | - |
|  |  | rs17693963 | 27818144 | C/A | 2.8 x 10^-7^ | 20 |  | A/C | 0.673 | 0.673 |
|  |  | rs7776351 | 27834710 | T/C | 3.2 x 10^-7^ | 20 |  | - | - | - |
|  |  | rs12182446 | 27853717 | A/G | 4.8 x 10^-7^ | 20 |  | - | - | - |
| MHC region/PGBD1 | 6 | rs13211507 | 28365356 | C/T | 8.3 x 10^-11^ | 21 |  | - | - | - |
| MHC region/ NOTCH4 | 6 | rs3131296 | 32280971 | A/G | 2.3 x 10^-10^ | 21 |  | - | - | - |
|  |  |  |  |  |  |  |  |  |  |  |
|  |  |  |  |  |  |  |  |  |  |  |
| MHC region/ HLA-DQA1 | 6 | rs9272219 | 32710247 | T/G | 6.9 x 10^-8^ | 20,24 |  | G/T | 0.088 | 0.241 |
|  |  | rs9272535 | 32714734 | A/G | 8.9 x 10^-8^ | 20,24 |  | A/G | 0.099 | 0.301 |
|  |  | rs241427 | 32912392 | - | - | - |  | A/G | 0.00105 | 0.001263 |
| RELN | 7 | rs7341475 | 103192051 | G/A | 2.9 x 10^-5^ | 26 |  | A/G | 0.419 | 0.421 |
|  |  | rs362725 | 102992853 | - | - | - |  | C/T | 3.1 x 10^-4^ | 3.4 x 10^-4^ |
| SMARCA2 (Japan) | 9 | rs2066111 | 2063839 | G/A | 8.2 x 10^-5^ | 27 |  | - | - | - |
|  |  | rs3763627 | 2066174 | A/T | 1.2 x 10^-5^ | 27 |  | - | - | - |
|  |  | rs3793490 | 2085958 | T/G | 3.0 x 10^-6^ | 27 |  | A/C | 0.771 | 0.773 |
|  |  | rs2296212 | 2181309 | G/C | 5.8 x 10^-5^ | 27 |  | - | - | - |
| PLAA | 9 | rs7045881 | 26925996 | T/A | 2.1 x 10^-6^ | 22 |  | A/T | 0.095 | 0.983 |
| ANK3 | 10 | rs10761482 | 61755343 | C/T | 7.7 x 10^-6^ | 22 |  | C/T | 0.495 | 0.968 |
| Intergenic region on 11p14.1 | 11 | rs10835482 | 29079278 | - | - | - |  | C/T | 1.9 x 10^-4^ | 2.4 x 10^-4^ |
|  |  | rs12366025 | 29105466 | - | - | - |  | C/T | 1.0 x 10^-4^ | 1.1 x 10^-4^ |
|  |  | rs1552717 | 29115071 | - | - | - |  | A/T | 5.2 x 10^-4^ | 6.6 x 10^-4^ |
|  |  | rs1907605 | 29115083 | - | - | - |  | A/G | 5.6 x 10^-4^ | 7.1 x 10^-4^ |
|  |  | rs1602565 | 29118712 | C/T | 2.99 x 10^-6^ | 18 |  | C/T | 3.2 x 10^-4^ | 3.9 x 10^-4^ |
|  |  | rs2172225 | 29122778 | - | - | - |  | C/T | 2.7 x 10^-4^ | 3.3 x 10^-4^ |
|  |  | rs2958625 | 29124044 | - | - | - |  | A/C | 3.5 x 10^-4^ | 4.3 x 10^-4^ |
|  |  | rs10835491 | 29128455 | - | - | - |  | C/G | 5.1 x 10^-4^ | 6.1 x 10^-4^ |
|  |  | rs11030597 | 29147104 | - | - | - |  | C/T | 1.5 x 10^-4^ | 2.0 x 10^-4^ |
|  |  | rs11030606 | 29170988 | - | - | - |  | C/T | 6.8 x 10^-4^ | 8.9 x 10^-4^ |
| NRGN | 11 | rs12807809 | 124111495 | C/T | 2.4 x 10^-9^ | 21 |  | C/T | 0.309 | 0.837 |
|  |  | rs7116907 | 124307306 | - | - | - |  | C/G | 2.6 x 10^-4^ | 2.8 x 10^-4^ |
| Intergenic region on 16p13.2 | 16 | rs7192086 | 12969112 | T/A | 6.1 x 10^-6^ | 18 |  | A/T | 0.585 | 0.930 |
| ACSM1 | 16 | rs163260 | 20574283 | - | - | - |  | C/T | 0.00142 | 0.002 |
|  |  | rs433598 | 20587707 | T/C | 3.3 x 10^-6^ | 22 |  | C/T | 0.013 | 0.322 |
|  |  | rs163255 | 20597316 | - | - | - |  | C/T | 0.00116 | 0.003 |
|  |  |  |  |  |  |  |  |  |  |  |
|  |  |  |  |  |  |  |  |  |  |  |
| TCF4 | 18 | rs9646596 | 51200210 | A/G | 2.6 x 10^-7^ | 24 |  | - | - | - |
|  |  | rs17594526 | 51209236 | T/C | 1.3 x 10^-7^ | 24 |  | - | - | - |
|  |  | rs17594665 | 51214717 | A/G | 1.6 x 10^-7^ | 24 |  | A/G | 0.419 | 0.419 |
|  |  | rs17594721 | 51216890 | G/A | 3.7 x 10^-7^ | 24 |  | - | - | - |
|  |  | rs11152369 | 51217326 | C/A | 2.0 x 10^-7^ | 24 |  | - | - | - |
|  |  | rs17509991 | 51218182 | A/G | 2.9 x 10^-7^ | 24 |  | - | - | - |
|  |  | rs17510124 | 51219678 | C/T | 3.8 x 10^-7^ | 24 |  | - | - | - |
|  |  | rs7228846 | 51225616 | G/A | 2.8 x 10^-7^ | 24 |  | - | - | - |
|  |  | rs8089309 | 51226290 | C/A | 3.8 x 10^-7^ | 24 |  | - | - | - |
|  |  | rs1371832 | 51233234 | T/C | 4.0 x 10^-7^ | 24 |  | - | - | - |
|  |  | rs9960767 | 51306000 | A/C | 4.1 x 10^-9^ | 21 |  | - | - | - |
| CSF2RA, IL3RA | X,Y | rs4129148 | updated | C/G | 3.7 x 10^-7^ | 23 |  | - | - | - |
