# Supplementary material for: Genome-Wide Association Study of Treatment Refractory Schizophrenia in Han Chinese
Source: PLoS One. 2012 Mar 27;7(3):e33598. doi: 10.1371/journal.pone.0033598 (PMC3313922; doi:10.1371/journal.pone.0033598)
Supplement: Table S7 — Adjustment of the top SNPs for drinking and inclusion of 20 principal components as covariates in logistic regression. (DOCX) [file pone.0033598.s013.docx]

Supplementary Table 7 Adjustment of the top SNPs for drinking and inclusion of 20 principal components as covariates in logistic regression

| ch | SNP | position | *P* _trend_ | *P* _adjust for regular drinker_ | *P* _adjust for C1-C20_ |
| --- | --- | --- | --- | --- | --- |
| 1 | rs10218843 | 158892685 | 6.73 x 10^-6^ | 5.9956 x 10^-6^ | 3.8440 x 10^-6^ |
| 1 | rs11265461 | 158896767 | 5.90 x 10^-6^ | 5.1299 x 10^-6^ | 3.9250 x 10^-6^ |
| 4 | rs230529 | 103676448 | 1.07 x 10^-6^ | 1.4491 x 10^-6^ | 2.1114 x 10^-6^ |
| 4 | rs4699030 | 103722862 | 8.41 x 10^-7^ | 1.1574 x 10^-6^ | 1.7518 x 10^-6^ |
| 5 | rs461409 | 97957866 | 2.63 x 10^-6^ | 2.9604 x 10^-6^ | 4.3163 x 10^-6^ |
| 7 | rs12533497 | 91495608 | 1.04 x 10^-5^ | 1.1779 x 10^-5^ | 4.2614 x 10^-6^ |
| 7 | rs739617 | 111298102 | 1.46 x 10^-5^ | 1.3820 x 10^-5^ | 7.7557 x 10^-5^ |
| 7 | rs17158926 | 111298199 | 3.99 x 10^-5^ | 3.6166 x 10^-5^ | 1.6059 x 10^-4^ |
| 7 | rs17158930 | 111298374 | 3.08 x 10^-5^ | 2.7924 x 10^-5^ | 1.3371 x 10^-4^ |
| 8 | rs9314462 | 2501291 | 5.30 x 10^-5^ | 9.3198 x 10^-5^ | 9.0940 x 10^-5^ |
| 16 | rs9646303 | 86019470 | 1.15 x 10^-5^ | 7.2461 x 10^-6^ | 6.4178 x 10^-6^ |
| 19 | rs11673496 | 22581270 | 1.75 x 10^-5^ | 1.8694 x 10^-5^ | 2.2519 x 10^-5^ |
| 21 | rs13049286 | 42049868 | 1.23 x 10^-5^ | 2.8938 x 10^-5^ | 3.3972 x 10^-5^ |
| 21 | rs3827219 | 42053555 | 1.23 x 10^-5^ | 2.8429 x 10^-5^ | 2.6582 x 10^-5^ |
