# Supplementary material for: Genome-Wide Association Study of Treatment Refractory Schizophrenia in Han Chinese
Source: PLoS One. 2012 Mar 27;7(3):e33598. doi: 10.1371/journal.pone.0033598 (PMC3313922; doi:10.1371/journal.pone.0033598)
Supplement: Methods S1 — Principal component analysis using EIGENSTRAT. (DOCX) [file pone.0033598.s014.docx]

**Supplementary Methods 1:**

Principal component analysis using EIGENSTRAT

To select unlinked SNPs for principal component (PC) analysis, we first lined up the quality SNPs by physical position within each chromosome and selected the (1+ 4i)th SNPs, i = 0, 1, 2, etc.. Furthermore, we retained only one SNP in each linkage disequilibrium (LD) block in case two or more SNPs located in the same LD block. The LD blocks were estimated based on the CHB+JPT data set (hapmap3_r2_b36_fwd) of the International HapMap Project (www.hapmap.org), using Haploview with LD blocks defined by Gabriel et al. [1]. The final set comprised 76,673 SNPs.

We then performed PC analysis using EIGENSTRAT [2] using a default setting and obtained 20 principal components for further tests (Supplementary Table 7). Detections of population stratification and ethnicity outliers were carried out based on the principal components. For comparison, we also included the 20 principal components in logistic regressions as covariates to assess the association between the top SNPs (selected without PC correction) and the affected status.

References

1. Gabriel SB, Schaffner SF, Nguyen H, Moore JM, Roy J, et al. (2002) The structure of haplotype blocks in the human genome. Science 296: 2225-2229.

2. Price AL, Patterson NJ, Plenge RM, Weinblatt ME, Shadick NA, et al. (2006) Principal components analysis corrects for stratification in genome-wide association studies. Nat Genet 38: 904-909.
