# Supplementary material for: Genome-Wide Association Study of Treatment Refractory Schizophrenia in Han Chinese
Source: PLoS One. 2012 Mar 27;7(3):e33598. doi: 10.1371/journal.pone.0033598 (PMC3313922; doi:10.1371/journal.pone.0033598)
Supplement: Methods S2 — Analyses based on pair-wise identity-by-state (IBS) distance using Plink. (DOCX) [file pone.0033598.s015.docx]

**Supplementary Methods 2**

*Analyses based on pair-wise identity-by-state (IBS) distance using Plink*

Pair-wise identity-by-state (IBS) distance based on all 516,212 quality SNPs was used to identify pairs who might be a close relative to each other. Multidimensional scaling analysis and outlier detection was performed based on based on the pair-wise IBS distance. Permutation test for between group IBS differences was performed with a fixed 10,000 permutations. For the purpose of stratification effects between cases and controls, we reported the p-value of testing whether or not, on average, an individual was less similar to another phenotypically-discordant individual than would be expected by chance (denoted as T1 in PLINK). PLINK [1] was used to carry out the analysis.

Reference:

1. Purcell S, Neale B, Todd-Brown K, Thomas L, Ferreira MA, et al. (2007) PLINK: a tool set for whole-genome association and population-based linkage analyses. Am J

Hum Genet 81: 559-575.
