# Supplementary material for: Genome-Wide Association Study of Treatment Refractory Schizophrenia in Han Chinese
Source: PLoS One. 2012 Mar 27;7(3):e33598. doi: 10.1371/journal.pone.0033598 (PMC3313922; doi:10.1371/journal.pone.0033598)
Supplement: Methods S3 — Genomic Control. (DOCX) [file pone.0033598.s016.docx]

**Supplementary Methods 3**

Genomic Control

Procedures for genomic control were carried out according to methods by Devlin and Roeder [1] and by Zheng et al [2]. Variance inflation factor, lambda, was estimated for trend, allele-type, dominant, and recessive chi-square tests, based on all 694,436 quality SNPs. P-values with genomic control were calculated for comparison.

References:

1. Devlin B, Roeder K (1999) Genomic control for association studies. Biometrics 55:

997-1004.

2. Zheng G, Freidlin B, Gastwirth JL (2006) Robust genomic control for association studies. Am J Hum Genet 78: 350-356.
